# Effects of resistance training intensity on muscle quantity/quality in middle‐aged and older people: a randomized controlled trial

**DOI:** 10.1002/jcsm.12941

**Published:** 2022-02-20

**Authors:** Yuta Otsuka, Yosuke Yamada, Akifumi Maeda, Takayuki Izumo, Tomohiro Rogi, Hiroshi Shibata, Masahiro Fukuda, Takuma Arimitsu, Naokazu Miyamoto, Takeshi Hashimoto

**Affiliations:** ^1^ Institute for Health Care Science Suntory Wellness Ltd. Kyoto Japan; ^2^ National Institute of Health and Nutrition National Institutes of Biomedical Innovation, Health and Nutrition Tokyo Tokyo Japan; ^3^ Suntory Global Innovation Center Ltd. Research Institute Kyoto Japan; ^4^ Faculty of Sport and Health Science Ritsumeikan University Kyoto Japan; ^5^ Fukuda Clinic Osaka Japan; ^6^ Faculty of Health Care, Undergraduate Department of Human Health Hachinohe Gakuin University Hachinohe Japan; ^7^ Graduate School of Health and Sports Science Juntendo University Chiba Japan

**Keywords:** Resistance exercise, Magnetic resonance imaging, Dual‐energy X‐ray absorptiometry, Segmental bioelectrical impedance spectroscopy

## Abstract

**Background:**

A sarcopenia diagnosis is confirmed by the presence of low muscle quantity or quality under the 2018 revised definition by the European Working Group on Sarcopenia in Older People 2. Imaging methods [i.e. magnetic resonance imaging (MRI)], dual‐energy X‐ray absorptiometry (DXA), and bioelectrical impedance analysis are tools to evaluate muscle quantity or quality. The present study aimed to investigate whether and how low‐intensity and moderate‐intensity resistance training improved both muscle quantity and quality measured by MRI, DXA, and segmental bioelectrical impedance spectroscopy (S‐BIS) in middle‐aged and older people.

**Methods:**

A single‐blind, randomized, controlled trial was conducted. Community‐dwelling people aged 50–79 years were randomly allocated to no exercise (no‐Ex), low‐intensity exercise (low‐Ex), and moderate‐intensity exercise (moderate‐Ex) groups. Participants in the exercise groups performed resistance training for 24 weeks, with loads of 40% and 60% of one repetition maximum in the low‐Ex and moderate‐Ex groups, respectively. Cross‐sectional area (CSA), lean mass, and muscle electrical properties on S‐BIS were used to determine the effects of training interventions on muscle quantity and quality of the lower limbs.

**Results:**

Fifty participants (no‐Ex 17, age 63.5 ± 8.5 years, women 47.1%; low‐Ex 16, age 63.6 ± 8.1 years, women 50.0%; moderate‐Ex 17, age 63.5 ± 8.3 years, women 52.9%) completed the 24 week exercise intervention. For the primary outcome, significant intervention effects were found in thigh muscle CSA on MRI between the moderate‐Ex and no‐Ex groups (+6.8 cm^2^, *P* < 0.01). Low‐Ex for 24 weeks only increased quadriceps CSA (+2.3 cm^2^, *P* < 0.05). The per cent change of thigh muscle CSA (+7.0%, *P* < 0.01) after 24 week moderate‐Ex was higher than that of leg lean mass on DXA (+2.3%, *P* = 0.088). Moderate‐Ex for 24 weeks also improved S‐BIS electrical properties related to muscle quantity and quality, including the intracellular resistance index (+0.1 cm^2^/Ω, *P* < 0.05), membrane capacitance (+0.7 nF, *P* < 0.05), and phase angle (+0.3 deg, *P* < 0.05); their changes were positively correlated with that of thigh muscle CSA (*P* < 0.01).

**Conclusions:**

Resistance exercise with moderate intensity improved muscle quantity and quality measured by MRI and S‐BIS, whereas that with low intensity only increased muscle quantity in middle‐aged and older people. The comparisons among the responses to exercise between the assessment methods indicate the greater value of MRI and S‐BIS to measure changes of muscle quantity and quality than of lean mass measured by DXA for assessing the local effects of resistance training.

## Introduction

Sarcopenia, characterized by age‐related declines in skeletal muscle mass and strength and/or physical function,^1^ has become a worldwide social issue, because sarcopenia induces high risks of frailty, mobility limitation and mortality in the older population. A sarcopenia diagnosis is confirmed by the presence of low muscle quantity or quality under the 2018 revised definition by the European Working Group on Sarcopenia in Older People 2 (EWGSOP2).^2^ In the EWGSOP2 document, imaging methods [i.e. magnetic resonance imaging (MRI) and computed tomography (CT)], dual‐energy X‐ray absorptiometry (DXA), and bioelectrical impedance analysis (BIA) are described as tools to assess skeletal muscle mass (quantity). MRI and CT are considered to be gold standards for non‐invasive assessment of muscle quantity. However, they are not commonly used in primary care because of high equipment costs, lack of portability, and the requirement for highly trained experts to use them. DXA is a more widely used device to determine muscle quantity and is presently favoured by some clinicians and researchers for measuring muscle mass. An advantage of DXA is that it can provide a reproducible estimate of lean mass, but disadvantages are that the DXA device is not portable for use in the community and exposes patients to X‐rays. DXA measurements can also be affected by the hydration status of the patient.^2^

The EWGSOP2 also mentioned ‘muscle quality’, referring to both microscopic and macroscopic changes in muscle architecture and composition, and to muscle function delivered per unit of muscle mass.^2^ High‐resolution imaging tools such as MRI or CT have been used to assess muscle quality in research settings. For instance, the infiltration of fat into muscle and the attenuation of the muscle have been used to determine muscle quality.^3^ Alternatively, muscle quality has been assessed by BIA‐derived phase angle measurements and/or other electrical properties of the limbs measured by segmental bioelectrical impedance spectroscopy (S‐BIS).^4^, ^5^, ^6^, ^7^ BIA or S‐BIS equipment is affordable, widely available, and portable. However, this equipment does not measure muscle mass directly, but it instead derives an estimate of muscle quantity and/or quality based on electrical conductivity. Therefore, examining the effects of interventions on phase angle and other electrical properties is very attractive for research and clinical practice.

Resistance exercise training is recognized as the most reliable approach for maintaining muscle quantity and quality.^2^ The American College of Sports Medicine recommends that high‐intensity resistance exercise, about 80% 1‐repetition maximum (1‐RM), is needed to achieve maximum effects for muscle hypertrophy in not only young adults, but also elderly persons.^8^, ^9^ However, there are some problems with respect to continuity and safety when elderly persons train with a high‐intensity protocol.^10^ Meanwhile, a systematic review of dose–response relationships of resistance training in muscle morphology of healthy elderly persons showed that exercise protocols with less than 80% 1‐RM intensity are also effective.^11^ Indeed, low‐intensity (40% 1‐RM) resistance exercise for 52 weeks showed an increase in cross‐sectional area (CSA) of Types 1 and 2 muscle fibres in biopsies of elderly women.^12^ Moreover, moderate‐intensity (60% 1‐RM) resistance training for 12 weeks also increased midthigh muscle CSA measured by MRI.^13^ These reports indicated that the improvement of muscle quantity by low‐intensity or moderate‐intensity exercise training could be detected by measuring CSA, whereas little has been reported about the intensity‐dependent effects on muscle quality. Therefore, the appropriate combination of exercise intensity and period to increase both muscle quantity and quality safely in elderly persons needs to be clarified with the various methods recommended by EWGSOP2.

In the present randomized, controlled trial, the aim was to investigate whether 24 week low‐intensity and moderate‐intensity resistance training improved muscle quantity and quality in middle‐aged and older people. The present study also aimed to compare different assessments of the effects of resistance training on muscle quantity and quality, as described in EWGSOP2 including MRI, DXA, and S‐BIS. The exercise protocol in this study consisted of several training sessions focused on thigh muscle, which decreases markedly with age in sarcopenia.^14^

## Methods

### Study design

A randomized, single‐blind, no treatment controlled, parallel group intervention was conducted to evaluate the effects of two types of resistance exercise training on muscle quantity and quality. Community‐dwelling Japanese persons living in Osaka were recruited, and 147 participants were screened; 61 were randomly allocated to the no‐exercise (no‐Ex), low‐intensity exercise (low‐Ex), and moderate‐intensity exercise (moderate‐Ex) groups. Randomization was based on dynamic allocation to achieve balance among the groups with regard to age, sex, and lower muscle mass using a spreadsheet programme with the RAND function (Microsoft Excel 2013, Microsoft Corporation, Redmond, WA, USA). Lower muscle mass was measured by a multiple‐frequency, body composition meter MC‐780A (Tanita, Tokyo, Japan).^15^ The randomization codes for participants and groups were each held in sealed opaque envelopes by two different individuals who were not involved in this study. A research nurse opened the envelopes when all data were collected and analysed. This study was designed as a single‐blind, randomized, controlled trial in which researchers involved in the measurements of outcomes were blinded to the randomization assignment.

Participants received interventions for 24 weeks between May and November 2018. Blood and urine were sampled following overnight fasting for safety assessment during the screening period, at baseline, at 12 weeks, and at 24 weeks. MRI, DXA, and S‐BIS were performed at baseline, 12 weeks, and 24 weeks following the intervention. Participants recorded changes of physical condition and habituation, and daily walk steps using a PD‐635 pedometer (Tanita) in the study diary to calculate average walk steps every 4 weeks. They were instructed not to change their lifestyles, including exercise habits during the intervention period. This study was registered in the University Hospital Medical Information Network (UMIN) Clinical Trial Registry (UMIN000032135).

### Population

The participants were community‐dwelling Japanese men and women aged 50 to 79 years, who did not exercise regularly more than twice a week and more than 30 min per times in the past year before starting the screening. Exclusion criteria were the presence of disease affecting the locomotor organs; presence of cardiovascular disease limiting exercise intervention; a history of serious disorders and clinically significant systemic diseases; problems receiving exercise intervention; planning weight loss; previous experience with high‐intensity exercise, such as bodybuilder or full marathon runner; an irregular lifestyle; heavy drinker; heavy smoker; consumption of drugs or supplements that affect efficacy evaluation; not capable of undergoing MRI measurements, such as having magnetic material or a tattoo in the body or claustrophobia; pregnant women; nursing mothers or women of child‐bearing potential; and the presence of any medical condition judged by the medical investigator to be incompatible with participation in the study.

### Outcomes

The primary outcome was the change in thigh muscle CSA over 24 weeks. Secondary outcomes were other CSAs, whole‐body lean mass, and electrical properties of the thigh measured by S‐BIS. Safety was assessed based on the incidence of side effects and adverse events among the groups during the 24 week intervention.

### Sample size calculations

In the absence of preliminary data to estimate the expected treatment difference, the sample size was set at 50 participants based on achieving a statistical power of 80% (type I error 5% using a two‐tailed test) to detect a clinically meaningful difference (effect size of 0.2) in the primary outcome, setting the number of groups and measurement number of 3 in the repeated‐measures analysis of variance (ANOVA) test. A sample size of 61 participants was required based on an expected drop‐out rate of 20%.

### Magnetic resonance imaging measurements

To assess muscle CSA, a 3.0‐T MR system (MAGNETOM Skyra, Siemens Healthineers, Erlangen, Germany) was used to obtain a series of axial slices from the superior border of the patella to the greater trochanter including the rectus femoris muscle. From the 10‐mm‐thick slices, the images of the right midthigh at the centre of the end‐to‐end images were analysed to measure thigh, quadriceps, vastus lateralis, and subcutaneous CSA by SliceOmatic Ver 4.3 software (TomoVision, Magog, Canada). Moreover, manual range selection was used to measure the intermuscular adipose tissue (IMAT) of CSA in thigh, according to the procedure of Mizuno *et al*.^16^

### Dual‐energy X‐ray absorptiometry measurements

Dual‐energy X‐ray absorptiometry (Lunar iDXA; GE Healthcare United Kingdom Limited, Buckinghamshire, UK) was used for whole‐body composition assessment. All measurements were performed by a medical technologist of Ritsumeikan University. Lean mass was obtained from the whole body and leg and arm regions, and bone mineral density was obtained from the whole body.

### Segmental bioelectrical impedance spectroscopy measurements

According to the method described previously,^4^, ^5^, ^6^, ^7^ bioelectrical impedance was measured using SFB7, ImpediMed (Pinkenba, QLD, Australia) with disposable tab‐type monitoring electrodes (2 × 2 cm, Red Dot, 3M, St. Paul, MN, USA). Impedance of the right upper leg segment was measured to place an injecting electrode on the right side of the body on the dorsal surface of the foot, proximal to the second and third metatarsophalangeal joints and on the dorsal surface of the hand, proximal to the second and third metacarpal‐phalangeal joints, while a sensing electrode was placed on right side of the body on the articular cleft between the femoral and tibial condyles and the anterior superior iliac spine. Segment length (L) was calculated from the anterior superior iliac spine of the femur to the lateral tibial malleolus. Resistances at zero (R_0_) and infinity (R_∞_) frequencies were determined by extrapolation after fitting the spectrum of bioimpedance data to the Cole–Cole model using Bioimp software (ImpediMed), and R_I_ was calculated using 1/[(1/R_∞_) − (1/R_0_)]. The intracellular impedance index, extracellular resistance index, resistance ratio of intracellular to extracellular fluid and skeletal muscle mass of the whole body were calculated as L^2^/R_I,_ L^2^/R_0_, and R_0_/R_I_, respectively. The membrane capacitance, characteristic frequency, and phase angle were also calculated from the Cole–Cole model.^7^

### Handgrip strength measurements

Handgrip strength was measured using a Handgrip Dynamometer (MG4800, CHARDER Electronic, Taichung City, Taiwan) to assess upper muscle strength. Both hands were evaluated twice, and the larger values were used to determine the maximum handgrip strength in each hand and calculate the average of both hands.

### Exercise intervention

The resistance exercise programmes were conducted every Monday, Wednesday, and Friday for 24 weeks; this training frequency and period were generally shown to be effective to achieve muscle hypertrophy.^11^ Before starting training, the nurse checked body temperature, blood pressure, and physical condition, and then the doctor decided whether participation was possible. A well‐trained instructor paired the participants and supervised all training sessions to check safety and accuracy. The 40 min programme constituted 30 min resistance training using a machine, with a 5 min warm‐up and a 5 min cool‐down. Because the focus was on the thigh muscle in this study, resistance training included four types of machine training: leg extension, leg curl, leg press, and chest press. The training weight was 40% 1‐RM in the low‐Ex group and 60% 1‐RM in the moderate‐Ex group. All training was 3 sets of 14 repetitions, according to the method descried by Taaffe *et al*.^12^ The rest period between sets was 2 min, which was often chosen in several studies,^11^ except for leg extension, which had a rest period of 2.5 min including the time to set the belt to ensure a safe posture. Determination of 1‐RM was repeated every 4 weeks to adjust the training weight, while during the first 2 weeks of the 24 week period, instructors trained participants about the appropriate forms, with gradually increasing intensity dependent on each subject. For safety, the 1‐RM test was performed with the indirect method where the maximum weights to complete 8 to 12 repetitions were measured and converted to the values of 1‐RM: 8 repetitions to 80%, 10 repetitions to 75%, and 12 repetitions to 70% 1‐RM.^17^ Attendance and numbers of sets completed in the logs of each participant were recorded by instructors to calculate the attendance rates. In the no‐Ex group, the participants did not perform any exercise sessions during the intervention period. The participants were instructed to keep their lifestyles the same as before starting, when marked changes in daily walk steps and exercise activities were found in the study diary every week.

### Nutritional assessments

Nutritional intakes were assessed using a 3 day dietary record^18^ that was completed over three continuous days before the time for other assessments. The participants completed the records of eating foods at breakfast, lunch, and dinner, and they were checked by assessors to calculate total energy, protein, fat, and carbohydrate per day using the Super Nutrition Calculation System HealthyMaker Pro 501 (Mushroomsoft, Okayama, Japan).

### Statistical analysis

Efficacy assessment was performed by a per‐protocol set analysis. To compare baseline data among the groups, one‐way analysis of variance for quantitative variables and the chi‐squared test for categorical variables were used. Differences between interventions over time were analysed by two‐way repeated‐measures ANOVA. When a significant group × time interaction was observed, actual values and changes from 0 weeks to 12 or 24 weeks among the groups were compared by Tukey's test, and actual values at 0 weeks and 12 or 24 weeks in each group were compared by Dunnett's test. Only for 1‐RM tests, actual values and changes from 0 weeks at each time points were compared between the low‐Ex and moderate‐Ex groups by Student's *t* test. Change rates from 0 to 24 weeks were compared among the groups by Tukey's test. Further subgroup analysis by sex and age was conducted, and two‐way ANOVA [group × sex (men, women) and group × age (50–59, 60–69, 70–79 years)] of change rates of muscle quantity and quality during the 24 week intervention was performed. Correlation analysis of muscle quantity and quality was performed to determine Pearson's correlation coefficients. Safety assessment was performed with the full analysis set. The incidence of side effects and adverse events was compared among groups by Bonferroni corrections after Fisher's exact test. *P* values of 0.05 or 0.01 were considered significant. Statistical analyses were carried out using IBM SPSS Statistics for Windows, Version 22.0 (IBM Corporation, Armonk, NY, USA) and JMP, Version 15.2 (SAS institute Inc, Cary, NC, USA).

## Results

### Enrolment and baseline data

**Figure 1:**
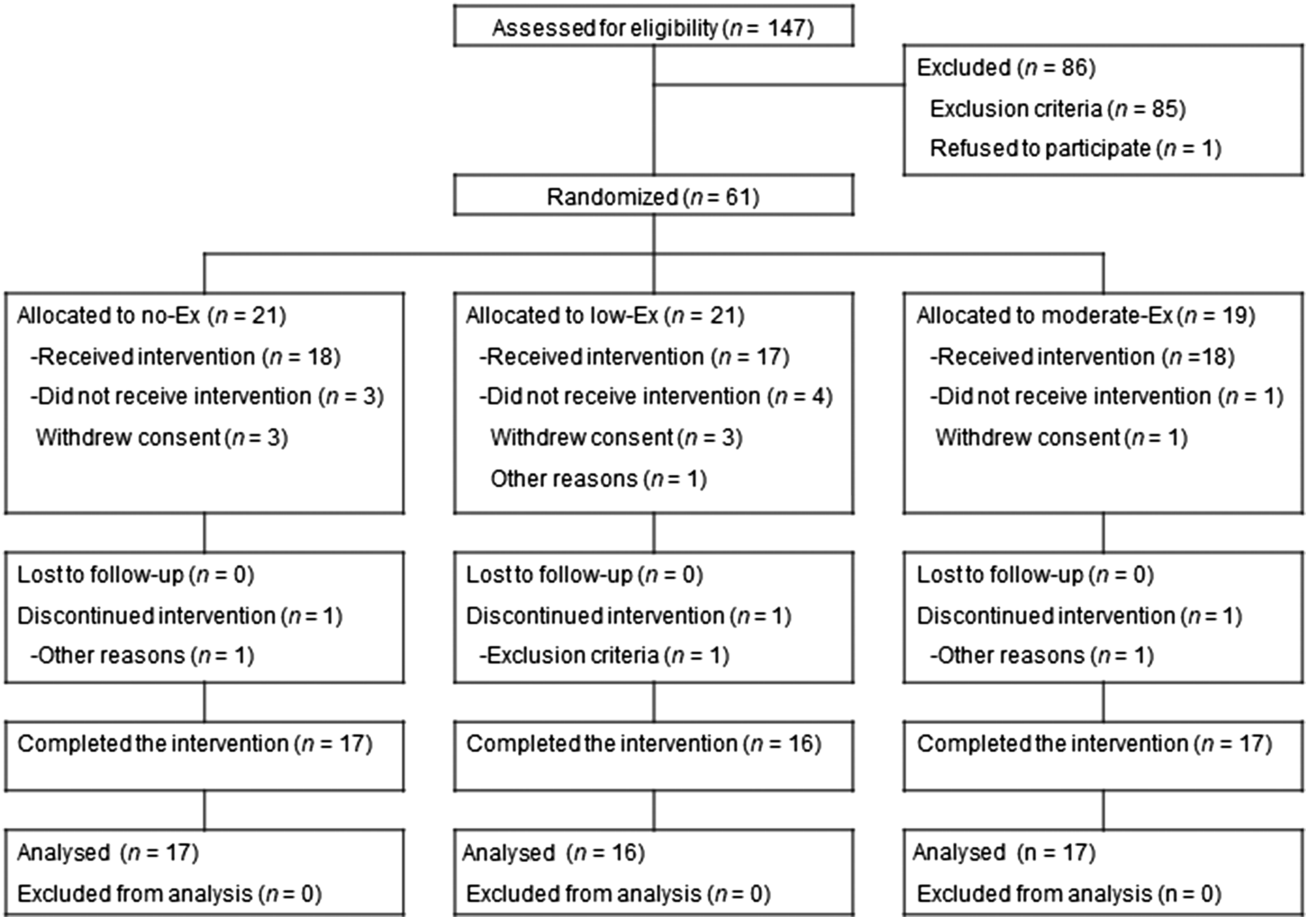
Flowchart of this study. Of 147 participants, 61 were randomly allocated to the no‐exercise (no‐Ex), low‐intensity exercise (low‐Ex), and moderate‐intensity exercise (moderate‐Ex) groups.

A total of 147 participants, 50–79 years old, were screened, 61 of whom were enrolled and randomly allocated to the three groups. Eight participants withdrew consent prior to the intervention or were unable to receive the interventions for other reasons. Fifty‐three participants began (*n* = 18 no‐Ex group; *n* = 17 low‐Ex group; *n* = 18 moderate‐Ex group) the interventions and were involved in the safety assessment (the full analysis set population). Three participants discontinued the interventions for various reasons. Fifty participants completed the 24 week intervention period, and the per‐protocol set population was used for efficacy assessment (*n* = 17 no‐Ex group; *n* = 16 low‐Ex group; *n* = 17 moderate‐Ex group; see flow chart in *Figure* 1). Mean attendance rates during the 24 week resistance exercise programme were 94.9% ± 5.4% in the low‐Ex group and 95.2% ± 4.0% in the moderate‐Ex group, with no significant difference between the groups (*P* = 0.861, data not shown). Baseline characteristics (age, sex, height, weight, thigh muscle CSA, and whole‐body lean mass) were similar among the three groups (*Table* 1). Daily walk steps and dietary intake of total energy, protein, fat and carbohydrate at baseline were also similar (*Table* S1), and there were no significant group × time interactions during the 24 week intervention (data not shown).

**Table 1:** Participants' baseline characteristics

	No‐Ex (*n* = 17)	Low‐Ex (*n* = 16)	Moderate‐Ex (*n* = 17)	*P* value
Age (years)^a^	63.5 ± 8.5	63.6 ± 8.1	63.5 ± 8.3	0.999
Sex (men/women)^b^	9/8	8/8	8/9	0.943
Height (cm)^a^	162.2 ± 8.3	159.7 ± 7.1	161.6 ± 10.8	0.720
Weight (kg)^a^	59.3 ± 12.6	59.7 ± 11.9	59.1 ± 11.6	0.991
Thigh muscle CSA (cm^2^)^a^	100.9 ± 21.9	102.5 ± 26.3	101.6 ± 26.0	0.982
Whole‐body lean mass (kg)^a^	41.0 ± 9.1	41.3 ± 9.1	40.9 ± 9.4	0.994
Hand grip strength (kg)^a^	31.4 ± 8.3	30.4 ± 10.4	29.3 ± 9.0	0.813

CSA, cross‐sectional area; Ex, exercise.

Values are expressed as mean ± standard deviation. There was no significant difference among the groups in baseline data.

^a^ ANOVA.

^b^ χ^2^ test.

### Effects of resistance training on muscle quantity, quality, and strength

The effects of resistance exercise for 24 weeks on CSAs in the upper legs, whole‐body lean mass, and S‐BIS properties in the thigh were examined (*Table* 2). During the 24 week intervention period, significant group × time interactions (*P* < 0.05) were found on MRI (thigh muscle CSA, quadriceps CSA, and vastus lateralis CSA), on DXA (whole‐body lean mass), and on S‐BIS measurement (intracellular resistance index, resistance ratio of intracellular to extracellular fluid, membrane capacitance, and phase angle). However, no significant group × time interactions were observed in MRI‐measured subcutaneous CSA, IMAT, and IMAT per thigh muscle CSA, DXA‐measured leg and arm lean masses, and hand grip strength. Significant differences were observed in changes of thigh muscle CSA over 24 weeks, the primary outcome, between the moderate‐Ex and no‐Ex groups (+6.8 cm^2^ vs. −1.1 cm^2^; *P* < 0.01), as well as at 12 week follow up (+1.9 cm^2^ vs. −2.5 cm^2^; *P* < 0.05). The moderate‐Ex group also showed significantly increased quadriceps and vastus lateralis CSA at 12 week follow‐up (+2.7 cm^2^ vs. −1.2 cm^2^; *P* < 0.01, and +1.3 cm^2^ vs. −0.7 cm^2^; *P* < 0.01) and 24 week follow‐up (+3.5 cm^2^ vs. −1.7 cm^2^; *P* < 0.01, and +2.4 cm^2^ vs. +0.1 cm^2^; *P* < 0.01) compared with no‐Ex, whereas only quadriceps CSA was increased at 24 week follow‐up in the low‐Ex group (+2.3 cm^2^ vs. −1.7 cm^2^; *P* < 0.05). For whole‐body lean mass measured by DXA, a significant difference was observed between the moderate‐Ex and no‐Ex groups in changes of whole‐body lean mass following 24 week intervention (+0.8 kg vs. 0 kg; *P* < 0.01), but not in leg and arm lean masses. Of the electrical properties measured by S‐BIS, changes of the resistance ratio (+0.01 vs. −0.02; *P* < 0.05), membrane capacitance (+0.4 nF vs. −0.6 nF; *P* < 0.05), and phase angle (+0.1 deg vs. −0.2 deg; *P* < 0.01) by 12 weeks were significantly greater in the moderate‐Ex group than in the low‐Ex group, and changes of phase angle (+0.1 deg vs. −0.2 deg; *P* < 0.05) in the moderate‐Ex group were also larger than those in the no‐Ex group. At 24 week follow‐up, significant differences were observed in changes of the intracellular resistance index (+0.1 cm^2^/Ω vs. −0.5 cm^2^/Ω; *P* < 0.05), membrane capacitance (+0.7 nF vs. −0.4 nF; *P* < 0.05), and phase angle (+0.3 deg vs. 0 deg; *P* < 0.05) between the moderate‐Ex and no‐Ex groups. There were no significant group × time interactions in bone mineral density and whole‐body skeletal muscle mass measured by DXA and S‐BIS, respectively (supporting information *Table* S2). In both the low‐Ex and the moderate‐Ex groups, 1‐RM of all training increased dependent on the training periods (*Table* 3). There was a significant difference only in the changes of 1‐RM in leg press after 20 weeks between the low‐Ex and moderate‐Ex groups, although most 1‐RM changes, except for leg curl, were higher in the moderate‐Ex group than in the low‐Ex group.

**Table 2:**
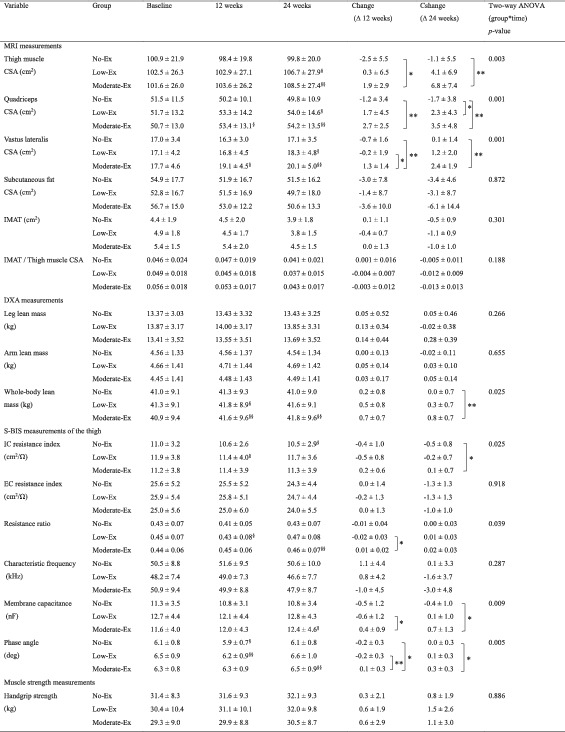
Muscle quantity, quality and strength in the groups during the intervention

CSA, cross‐sectional area; DXA, dual‐energy X‐ray absorptiometry; EC, extracellular; Ex, exercise; IC, intracellular; IMAT, intermuscular adipose tissue; MRI, magnetic resonance imaging; resistance ratio, resistance ratio of intracellular to extracellular fluid; S‐BIS, segmental bioelectrical impedance spectroscopy.

Values are expressed as mean ± standard deviation. For the no‐Ex (*n* = 17), low‐Ex (*n* = 16), and moderate‐Ex (*n* = 17) groups on MRI, DXA, and muscle strength measurements, and the no‐Ex (*n* = 16), low‐Ex (*n* = 15) and moderate‐Ex (*n* = 17) groups on S‐BIS measurements, where 2 participants were not included for analysis because of unmeasurable data, there were no significant differences among the groups at baseline (one‐way ANOVA).

* *P* < 0.05 compared among the groups (Tukey's test).

** *P* < 0.01 compared among the groups (Tukey's test).

^§^ *P* < 0.05 compared with values at baseline (Dunnett's test).

^§§^ *P* < 0.01 compared with values at baseline (Dunnett's test).

**Table 3:**
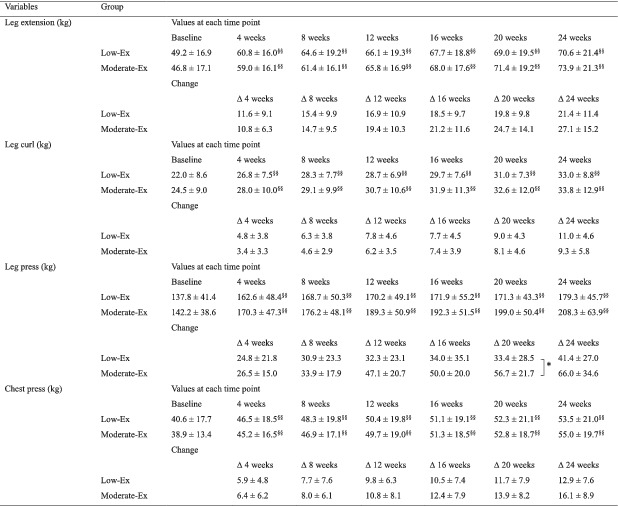
1‐RM in the groups during the intervention

Values are expressed as mean ± standard deviation. For the low‐Ex (*n* = 16), and moderate‐Ex (*n* = 17) groups on 1‐RM tests, there were no significant differences among the groups at baseline (Student's *t* test). 1‐RM, 1‐repetition maximum.

* *P* < 0.05 compared among the groups (Student's *t* test).

^§§^ *P* < 0.01 compared with values at baseline (Dunnett's test).

**Figure 2:**
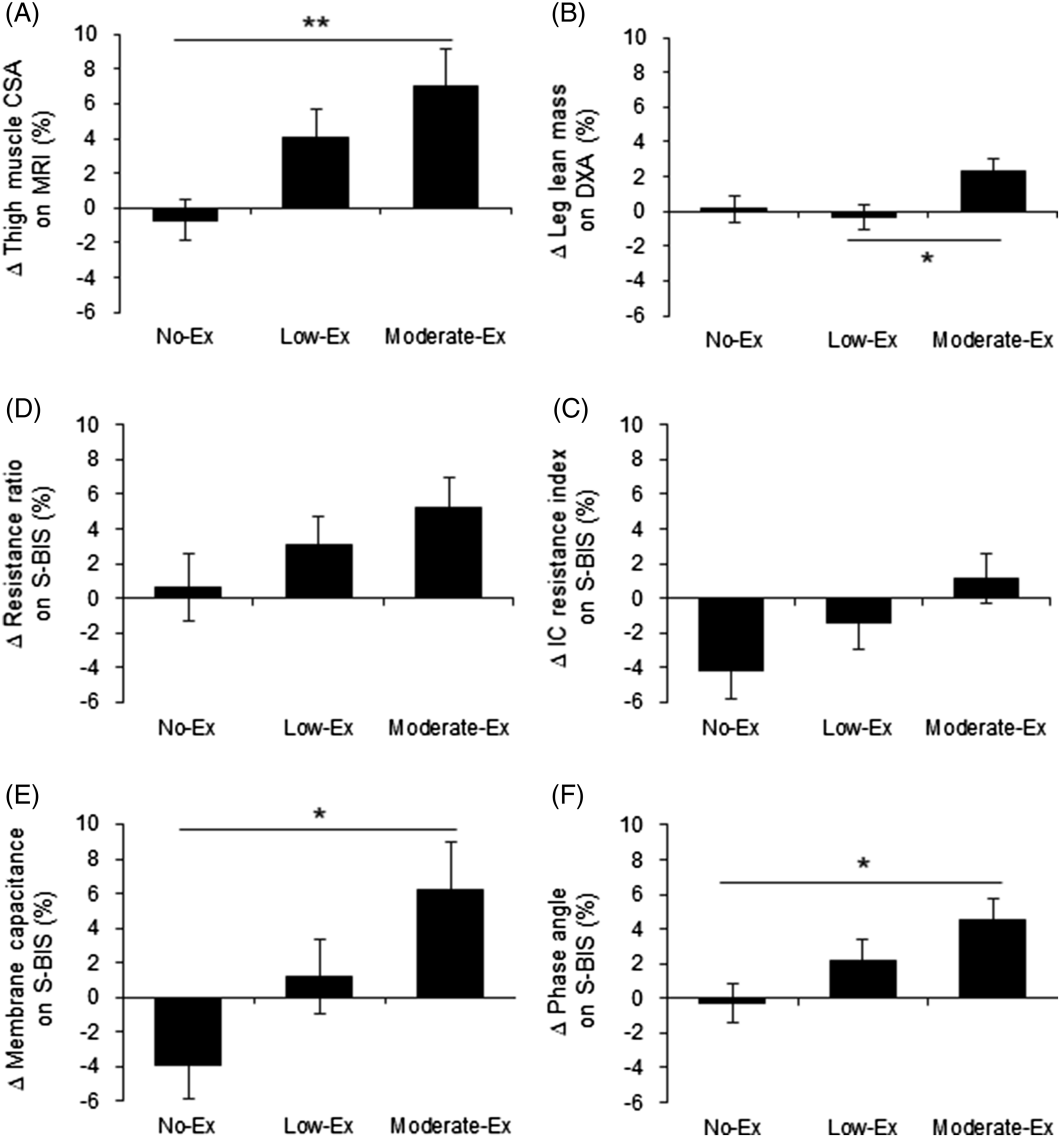
Change rates of muscle quantity and quality during the 24 week intervention. The change rates at 24 weeks from 0 weeks in muscle quantity [thigh muscle CSA on MRI (A), leg lean mass on DXA (B) and the intracellular resistance index on S‐BIS (C)] and in muscle quality [resistance ratio of intracellular to extracellular fluid (D), membrane capacitance (E), and phase angle (F)] are presented. Values are expressed as mean ± standard error. *P < 0.05 and **P < 0.01 compared among the groups (Tukey's test). CSA, cross‐sectional area; DXA, dual‐energy X‐ray absorptiometry; IC, intracellular; MRI, magnetic resonance imaging; S‐BIS, segmental bioelectrical impedance spectroscopy; resistance ratio, resistance ratio of intracellular to extracellular fluid.

In the change rates of muscle quantity during the 24 week intervention, thigh muscle CSA was significantly higher in the moderate‐Ex group than in the no‐Ex group (+7.0% vs. −0.7%; *P* < 0.01, *Figure* 2A), whereas leg lean mass (+2.3% vs. +0.1%; *P* = 0.088, *Figure* 2b) and the intracellular resistance index (+1.1% vs. −4.1%; *P* = 0.052, *Figure* 2C) were also higher in the moderate‐Ex group, but not significantly different compared with the no‐Ex group. For muscle quality, the moderate‐Ex group also showed significant increases in the change rates of membrane capacitance (+6.2% vs. −3.9%; *P* < 0.05, *Figure* 2E) and phase angle (+4.5% vs. −0.2%; *P* < 0.05, *Figure* 2F) at 24 week follow‐up, whereas the resistance ratio (+5.3% vs. +0.6%; *P* = 0.155, *Figure* 2D) was also higher in the moderate‐Ex group, but it was not significant compared with the no‐Ex group. Although two‐way ANOVA considering sex or age showed that there was a significant interaction effect of group × sex only for the change rate of leg lean mass, the change rates of muscle quantity and quality during the 24 week intervention differed between men and women (*Table* 4). Neither a significant main effect nor an interaction effect of group × age in any change rates was observed (*Table* S3).

**Table 4:** Change rates of muscle quantity and quality of men and women in each group during the 24 week intervention

Variable	Group	Men	Women	Two‐way ANOVA (*P* value)	
				Sex	Group × sex
Thigh muscle CSA (%)	No‐Ex	−1.5 ± 5.6	0.2 ± 4.1	0.253	0.205
	Low‐Ex	4.9 ± 9.0	3.2 ± 3.3		
	Moderate‐Ex	10.6 ± 10.5	3.9 ± 5.4		
Leg lean mass (%)	No‐Ex	1.6 ± 3.4	−1.5 ± 1.4	0.099	0.005
	Low‐Ex	1.2 ± 2.6	−1.8 ± 2.4		
	Moderate‐Ex	1.1 ± 2.9	3.4 ± 2.6		
IC resistance index (%)	No‐Ex	−5.9 ± 4.6	−1.9 ± 9.1	0.180	0.851
	Low‐Ex	−2.4 ± 5.6	−0.4 ± 6.3		
	Moderate‐Ex	0.3 ± 7.2	1.9 ± 5.1		
Resistance ratio (%)	No‐Ex	0.0 ± 7.7	1.4 ± 8.9	0.980	0.800
	Low‐Ex	4.1 ± 4.0	2.0 ± 8.3		
	Moderate‐Ex	5.0 ± 7.2	5.5 ± 7.0		
Membrane capacitance (%)	No‐Ex	−2.9 ± 7.6	−5.1 ± 9.3	0.619	0.615
	Low‐Ex	0.1 ± 7.6	2.5 ± 10.2		
	Moderate‐Ex	8.5 ± 9.8	4.2 ± 12.5		
Phase angle (%)	No‐Ex	−0.2 ± 4.1	−0.3 ± 5.6	0.848	0.951
	Low‐Ex	2.6 ± 3.7	1.6 ± 6.7		
	Moderate‐Ex	4.4 ± 4.7	4.6 ± 5.9		

Values are expressed as mean ± standard deviation for the no‐Ex (*n* = 9), low‐Ex (*n* = 8) and moderate‐Ex (*n* = 8) groups in men, and the no‐Ex (*n* = 8), low‐Ex (*n* = 8) and moderate‐Ex (*n* = 9) groups in women on muscle quantity and quality.

In the subgroup analysis by sex, the baseline characteristics were similar among the groups in both men and women (*Tables* S4 and S5). In men, there were significant group × time interactions in thigh muscle CSA, quadriceps CSA, vastus lateralis CSA, membrane capacitance and phase angle, and the changes were significantly higher in the moderate‐Ex group than in the no‐Ex group during the 12 or 24 week intervention (*Table* S6). On the other hand, in women, significant differences were observed only in the changes of leg lean mass among the three groups at 12 or 24 week follow‐up (*Table* S7). In both men and women, 1‐RM of all training increased during the 24 week intervention (*Tables* S8 and S9).

### Correlations among muscle quantity and quality

**Figure 3:**
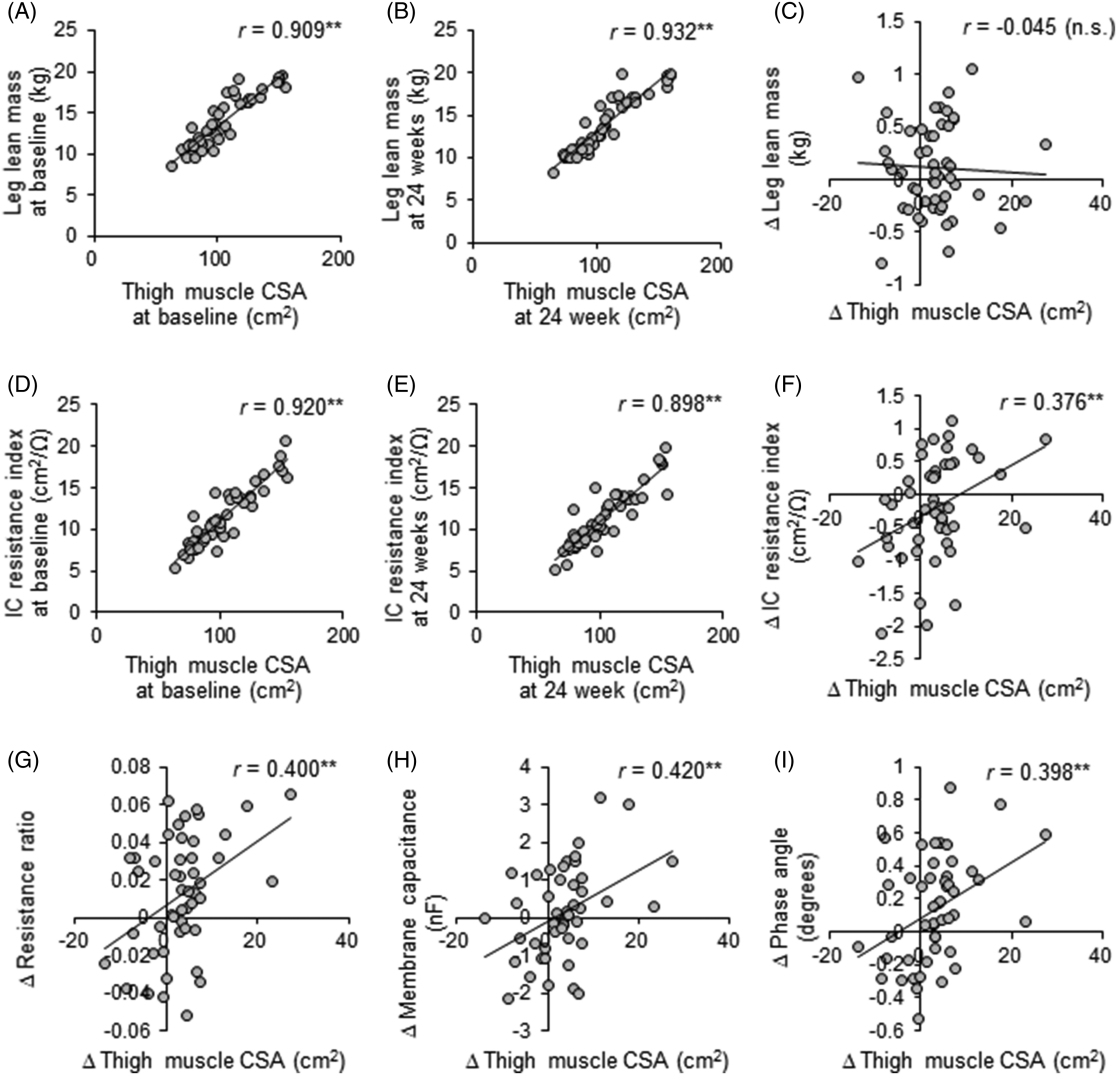
Relationships between thigh muscle CSA and leg lean mass on DXA or electrical properties on S‐BIS. Pearson's correlation co‐efficient (*r*) between thigh muscle CSA on MRI and leg lean mass on DXA (A–C) or the intracellular resistance index on S‐BIS (D–F) at baseline and at 24 weeks, and the changes during the 24 week intervention. Pearson's correlation coefficient (*r*) values between changes in thigh muscle CSA on MRI and the resistance ratio of intracellular to extracellular fluid (G), membrane capacitance (H), and phase angle (I) on S‐BIS during the 24 week intervention. **P < 0.01. CSA, cross‐sectional area; IC, intracellular; n.s., not significant; resistance ratio, resistance ratio of intracellular to extracellular fluid; S‐BIS, segmental bioelectrical impedance spectroscopy.

To analyse the relationships among each index of muscle quantity, correlation analysis was performed between thigh muscle CSA on MRI and other indices of quantity, leg lean mass on DXA or the intracellular resistance index on S‐BIS, because the change rate of thigh muscle CSA during the 24 week resistance training intervention was the highest, which means the change of thigh muscle CSA reflected the effects of resistance training the most. The correlations between thigh muscle CSA on MRI and leg lean mass on DXA (*Figure* 3A,B) or the intracellular resistance index (*Figure* 3D,E) on S‐BIS were high (*r* > 0.800; *P* < 0.01), both at baseline and at 24 week follow‐up. The change of the intracellular resistance index of S‐BIS (*r* = 0.376; *P* < 0.01, *Figure* 3F) was also positively correlated with the change of thigh muscle CSA on MRI during the 24 week intervention. However, the change of leg lean mass on DXA was not significantly correlated with the change of thigh muscle CSA (*r* = −0.045; *P* = 0.754, *Figure* 3C) and that of the intracellular resistance index (*r* = 0.111; *P* = 0.453, data not shown).

In the correlation between muscle quantity and quality, the changes in the resistance ratio (*r* = 0.400; *P* < 0.01, *Figure* 3G), membrane capacitance (*r* = 0.420; *P* < 0.01, *Figure* 3H), and phase angle (*r* = 0.398; *P* < 0.01, *Figure* 3I) were also significantly and positively correlated with the change of thigh muscle CSA. The change in subcutaneous fat CSA was not significantly correlated with the electrical properties (data not shown).

**Figure 4:**
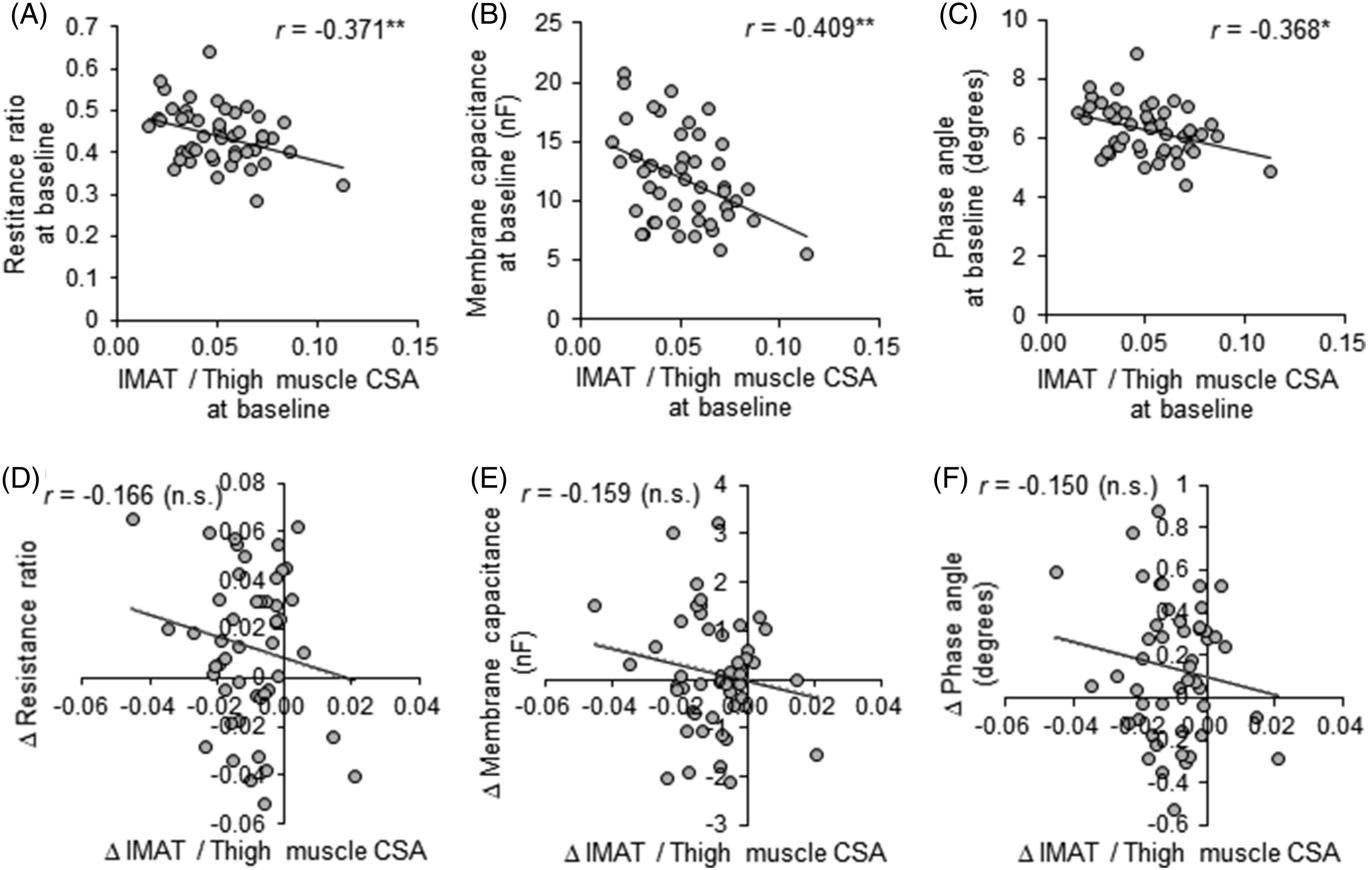
Relationships between the ratio of IMAT to thigh muscle CSA and electrical properties on S‐BIS. Pearson's correlation coefficient (*r*) values between the ratio of IMAT to thigh muscle CSA on MRI and the resistance ratio of intracellular to extracellular fluid (A, E), membrane capacitance (B, F), and phase angle (C, G) on S‐BIS at baseline, and the changes during the 24 week intervention. *P < 0.05 and **P < 0.01. IMAT, intermuscular adipose tissue; CSA, cross‐sectional area; n.s., not significant; resistance ratio, resistance ratio of intracellular to extracellular fluid; S‐BIS, segmental bioelectrical impedance spectroscopy.

In the correlation between muscle quality and quality, all electrical properties were significantly and negatively correlated with IMAT per thigh muscle CSA at baseline (*Figure* 4A–C), but there were no significant correlations between their changes during the 24 week intervention (*Figure* 4D–F).

### Safety

There were some adverse events with exercise, including three in the low‐Ex group (hip joint pain, foot sole pain, and high plasma CPK) and four in the moderate‐Ex group (right knee pain, knee pain, low back pain, and upper leg pain). However, there were no severe adverse events that required hospitalization, and there was no significant difference in the incidence of adverse events among groups.

## Discussion

This randomized, controlled trial showed improvements of muscle quantity and quality by resistance exercise training in middle‐aged and older people. Muscle quantity was increased in both the low‐Ex and the moderate‐Ex groups, with the effects dependent on the intensity and the period of exercise intervention, but muscle quality was improved only in the moderate‐Ex group for 24 weeks. Notably, the change rates of thigh muscle CSA on MRI were larger than those of leg lean mass on DXA, which indicated that CSA has higher sensitivity for exercise‐induced muscle quantity than leg lean mass in conditions of resistance training with a focus on the lower limb. Moreover, the changes in electrical properties on S‐BIS related to muscle quantity and quality were positively correlated with thigh muscle CSA. The present study suggests that MRI and S‐BIS would be useful for measuring muscle quantity and quality.

As recommended by American College of Sports Medicine, many studies have shown that high‐intensity resistance exercise with 70–80% 1‐RM has positive effects on muscle hypertrophy in elderly persons.^19^, ^20^ However, for older people, especially those with little physical activity, it is too difficult to continue these exercises in terms of safety and attitude.^10^ Moreover, as opposed to young people, a dose–response relationship of muscle protein synthesis to resistance exercise was not observed with more than 60% 1‐RM intensity exercise in elderly persons.^21^ In the present study, resistance exercises were set at 40% and 60% 1‐RM for 14 repetitions, 3 sets of each, in the low‐Ex and moderate‐Ex groups, respectively. The low‐intensity exercise for 24 weeks tended to increase most muscle quantity indices compared with the no‐Ex group, but a significant increase was observed only in the change of quadriceps CSA. On the other hand, the moderate‐Ex increased thigh, quadriceps, and vastus lateralis CSA, whole‐body lean mass, and the intracellular resistance index significantly (*Table* 2), with changes that were greater at 24 weeks than at 12 weeks. Consistent with the present data, Taaffe *et al*.^12^ demonstrated that resistance exercise with 40% 1‐RM intensity for 52 weeks increased muscle fibre CSA in elderly women, whereas that increase was more prominent with 80% 1‐RM intensity. In line with this observation, the present study also suggests that exercise with moderate‐intensity is more effective for muscle hypertrophy than low‐intensity exercise, and intervention for 24 weeks was recommended rather than 12 weeks in terms of the period. The present study also confirmed the increase of muscle strength in the lower limb as 1‐RM of each machine in both the low‐Ex and the moderate‐Ex groups during the 24 week intervention, although grip strength was slightly increased but not significantly, because the exercise protocol of this study was focused on the lower limbs.

MRI and DXA have been reported as tools to evaluate muscle quantity in EWGSOP2,^2^ but there are advantages and disadvantages with each measurement. DXA is more widely used by clinicians and researchers, although lean mass estimated by DXA may have some limitations for examining the muscle hypertrophy caused by resistance training exercise. In fact, the present study showed that the per cent change of thigh muscle CSA on MRI after 24 week resistance training exercise (+7.0%, *Figure* 2A) was more than triple the per cent change of leg lean mass on DXA (+2.3%, *Figure* 2B). This supports the results of previous studies.^22^, ^23^ The possible reasons are as follows: DXA is affected by hydration status,^2^ and it is insensitive to change of muscle attenuation and/or fat infiltration in skeletal muscle.^22^ In addition, thigh muscle CSA measured on MRI can reflect the change of muscle architecture in the horizontal plane of the legs well in resistance training exercise, but DXA using parasagittal beams from the front of the body may not be very sensitive to detect the muscle hypertrophy in the leg. The intracellular resistance index of S‐BIS also offers information about muscle quantity, which is distinguished from the effect of extracellular water in skeletal muscle composition.^7^ In the present results, moderate‐Ex significantly increased the intracellular resistance index compared with no‐Ex (*Table* 2). Notably, the change in thigh muscle CSA during the 24 week training intervention was positively correlated with the change in the intracellular resistance index, but not in leg lean mass on DXA, although both values at baseline and at 24 weeks were highly correlated with thigh muscle CSA (*Figure* 3A–F). Therefore, the intracellular resistance index on S‐BIS is highly sensitive for detecting the effects of resistance training on muscle quantity, like CSA on MRI.

Muscle quality is represented by not only muscle strength per unit of muscle mass, which is affected by several factors including changes of the neuromuscular junction, calcium homeostasis, and fibre type composition,^24^ but also by muscle composition, which can be measured by S‐BIS as changes in electrical properties with age including fibre atrophy, fibrosis, and intermuscular fat accumulation.^4^, ^5^, ^6^, ^7^ Age‐related changes in electrical properties on S‐BIS might reflect an age‐related decrease of muscle function rather than of leg lean mass.^7^ The electrical properties measured by S‐BIS include the resistance ratio of intracellular to extracellular fluid, membrane capacitance, the characteristic frequency, and the phase angle, which may reflect muscle quality with changing muscle composition.^7^ In the present study, the effects of low‐intensity and moderate‐intensity resistance exercise interventions on S‐BIS properties in the thigh were evaluated. The results showed that moderate‐intensity exercise, not low‐intensity exercise, significantly increased membrane capacitance and phase angle during the 24 week intervention (*Table* 2, *Figure* 2), and the changes were positively correlated with thigh muscle CSA (*Figure* 3H,I), but not subcutaneous fat CSA. The change in the resistance ratio of intracellular to extracellular fluid was also positively correlated with the change of thigh muscle CSA (*Figure* 3G), although there was no significant difference between the no‐Ex and Ex groups (*Table* 2, *Figure* 2). These findings indicate that S‐BIS properties can be used to detect improvements in muscle quality by resistance training. Moreover, changes in S‐BIS properties parallel those in muscle quantity defined as muscle CSA on MRI, and not changes in surrounding subcutaneous fat. Of note, there were significant negative correlations between the ratio of IMAT to thigh muscle CSA and S‐BIS properties at baseline (*Figure* 4A–C), which indicated that muscle composition measured by S‐BIS reflected the direct evaluation of muscle fat infiltration by MRI, thereby reflecting muscle quality. However, both low‐intensity and moderate‐intensity exercise slightly decreased the ratio of IMAT to thigh muscle CSA, but not significantly compared with no exercise (*Table* 2), with no correlations between it and S‐BIS properties during the 24 week intervention (*Figure* 4D–F). In line with the present results, resistance exercise did not reduce IMAT in older adults,^25^, ^26^ although endurance exercise did improve muscle fat infiltration.^27^, ^28^ Taken together, muscle composition measured by S‐BIS reflecting muscle quality would be responsive to resistance exercise, because electrical properties on S‐BIS were reflected by not only changes of intramuscular fat, but also water contents and resistance of the cell membrane. The phase angle may be useful as one of the sarcopenic markers due to the positive associations with muscle mass and function,^29^ so that an increased phase angle would be related to the preventive effect of interventions on sarcopenia. Previous reports showed that resistance exercise improved the phase angle in the whole body,^30^, ^31^, ^32^, ^33^ but there were no reports evaluating regional phase angles, as in the present study. Moreover, the present study was the first to report an increase in membrane capacitance with resistance exercise. A decrease in the capacitance was caused by a reduction in membrane content, which would be associated with muscle fibre loss.^7^ The improvement of these properties would lead to increased muscle function through the independent mechanism of muscle hypertrophy. Although further investigations of the molecular mechanism are needed, the present study provides evidence that moderate‐intensity exercise for 24 weeks improves the muscle profile composition measured by S‐BIS, which would be more useful for assessments of muscle quality than IMAT measured by MRI. S‐BIS is a lower cost and easier to perform test than MRI; thus, S‐BIS would be appropriate for daily‐living or primary evaluation of muscle quantity and quality in the clinical setting.

The present study had some limitations. First, significant differences were observed only in changes but not in actual values at 12 or 24 weeks. The reason was that baseline characteristics of muscle properties were different in individuals and were affected by sex and age, and the present study had a relatively small sample size that included men and women. However, the present study indicated sex differences not only in baseline characteristics but also in the effects of resistance training on muscle quantity and quality (*Table* 4). In fact, thigh muscle CSA, as well as other CSAs, was significantly increased by moderate‐Ex for 24 weeks compared with no‐Ex only in men, not in women (*Table*s S6 and S7). Similarly, membrane capacitance was also improved only in men during the 24 week intervention of moderate‐Ex (*Table*s S6 and S7). These differences would be caused by the higher absolute amount of training intensity and greater increase of 1‐RM changes in all resistance training in men than in women (*Table*s S8 and S9). Moreover, of the 25 female participants, 24 were of postmenopausal; thus, it is possible that the reduction in the effects of exercise in women might be caused by hormonal changes in menopause which affects exercise tolerance.^34^ These findings indicate that different exercise prescriptions are needed for each sex to improve muscle quantity and quality. Second, there was a lack of follow‐up evaluation to determine the longevity of the changes in muscle quantity and quality. During the 24 week intervention period, muscle quantity and quality were more improved by a higher intensity resistance exercise, but exercise of 40%‐1RM intensity, which was easier to continue, could also increase muscle quantity. Therefore, low‐intensity exercise could contribute to improve sarcopenia‐related reductions in quality of life, if the subjects continued this exercise. Further studies are needed to clarify the long‐term effects of the exercise protocols in this study. Conversely, the strengths of this study include the high attendance rates during the 24 week resistance exercise programme. Moreover, there were no severe adverse events, and no significant difference in the incidence of adverse events among groups. Therefore, both low‐intensity and moderate‐intensity exercise regimens in this study were considered easy and safe to continue under the conditions described here.

In conclusion, a higher intensity of resistance exercise was more effective for improving muscle quantity, and in particular low‐intensity exercise for 24 weeks could also increase muscle quantity. As for muscle quantity, thigh muscle CSA on MRI had a higher sensitivity for detection of changes due to resistance training than leg lean mass on DXA, and the intracellular resistance index on S‐BIS was also increased by moderate‐intensity exercise. Changes in the intracellular resistance index were positively correlated with those of thigh muscle CSA. These findings underscore the higher value of MRI and S‐BIS to measure changes of muscle quantity compared with lean mass measured by DXA for assessing the local effects of resistance training. Moreover, moderate‐intensity exercise for 24 weeks also improved electrical properties on S‐BIS, and significant correlations between the changes in CSA and these properties were found. These exercise‐induced changes would contribute to improve muscle composition as one aspect of muscle quality. Therefore, moderate‐intensity resistance exercise training at 60% 1‐RM is enough to improve both muscle quantity and quality in middle‐aged and older people. Additional studies are needed to investigate whether the exercise‐induced changes in muscle quantity and quality contribute to the improvement of physical function and quality of life in elderly people. Furthermore, a recent guideline suggests that the combination of exercise and nutrition is recommended for the prevention of sarcopenia.^35^ Further investigations are warranted to examine whether resistance exercise training, considering appropriate intensity, combined with nutrition could be more effective therapy for sarcopenia.

## Funding

This study was totally funded by Suntory Wellness Ltd.

## Conflict of interests

This work was supported by Suntory Wellness Ltd. Y. O., T. I., T. R., and H. S. are employees of Suntory Wellness Ltd., which manufactures and sells health food products. A. M. was an employee of Suntory Global Innovation Center Ltd. Other authors have no conflicts of interest to disclose.

## Ethics statements

The Ethics Committee of The Fukuda Clinic and Ritsumeikan University approved the study protocol in compliance with the Declaration of Helsinki. All patients gave their informed consent prior to their inclusion in the study.

## Supporting information

Table S1. Participants' baseline physical activity and dietary intake
Table S2. Bone mineral density and whole‐body skeletal muscle mass in the groups during the intervention
Table S3. Change rates of muscle quantity and quality by age in each group during the 24‐week intervention.
Table S4. Baseline characteristics in male participants
Table S5. Baseline characteristics in female participants
Table S6. Muscle quantity, quality and strength in men by groups during the intervention
Table S7. Muscle quantity, quality and strength in women by groups during the intervention
Table S8. 1‐RM in men by groups during the intervention
Table S9. 1‐RM in women by groups during the intervention
