# Supplementary material for: Effects of resistance training intensity on muscle quantity/quality in middle‐aged and older people: a randomized controlled trial
Source: J Cachexia Sarcopenia Muscle. 2022 Feb 20;13(2):894–908. doi: 10.1002/jcsm.12941 (PMC8977953; doi:10.1002/jcsm.12941)
Supplement: Supplementary file 1 — Table S1. Participants' baseline physical activity and dietary intake Table S2. Bone mineral density and whole‐body skeletal muscle mass in the groups during the intervention Table S3. Change rates of muscle quantity and quality by age in each group during the 24‐week intervention. Table S4. Baseline characteristics in male participants Table S5. Baseline characteristics in female participants Table S6. Muscle quantity, quality and strength in men by groups during the intervention Table S7. Muscle quantity, quality and strength in women by groups during the intervention Table S8. 1‐RM in men by groups during the intervention Table S9. 1‐RM in women by groups during the intervention [file JCSM-13-894-s001.docx]

**Supplementary Table 1. Participants’ baseline physical activity and dietary intake**

|  | No-Ex  (n = 17) | Low-Ex  (n = 16) | Moderate-Ex  (n = 17) | *p*-value |
| --- | --- | --- | --- | --- |
| Physical activity | | | | |
| Walk steps (steps/day) | 7852.6 ± 3251.5 | 8026.6 ± 2151.7 | 7588.9 ± 3049.7 | 0.907 |
| Dietary intake | | | | |
| Energy (kcal/day) | 1751.6 ± 441.0 | 1772.9 ± 255.4 | 1808.8 ± 197.3 | 0.869 |
| Protein (g/day) | 67.1 ± 13.1 | 69.1 ± 8.9 | 66.3 ± 11.7 | 0.773 |
| Fat (g/day) | 57.1 ± 20.8 | 62.7 ± 17.1 | 59.8 ± 13.5 | 0.655 |
| Carbohydrate (g/day) | 236.4 ± 58.1 | 226.7 ± 40.5 | 242.2 ± 29.5 | 0.603 |

Values are expressed as mean ± standard deviation. There was no significant difference among the groups in baseline data (ANOVA). Ex, exercise.

**Supplementary Table 2. Bone mineral density and whole-body skeletal muscle mass in the groups during the intervention**

| Variable | Group | Baseline | 12 weeks | 24 weeks | Change  (Δ 12 weeks) | Change  (Δ 24 weeks) | Two-way ANOVA  (group*time)  *p*-value |
| --- | --- | --- | --- | --- | --- | --- | --- |
| DXA measurements | | | | | | | |
| Whole BMD  (g/cm^2^) | No-Ex | 1.11 ± 0.12 | 1.11 ± 0.12 | 1.10 ± 0.12 | 0.000 ± 0.008 | ‐0.004 ± 0.008 | 0.724 |
|  | Low-Ex | 1.09 ± 0.14 | 1.09 ± 0.13 | 1.09 ± 0.14 | 0.001 ± 0.011 | ‐0.001 ± 0.009 |  |
|  | Moderate-Ex | 1.10 ± 0.13 | 1.10 ± 0.13 | 1.10 ± 0.13 | ‐0.002 ± 0.012 | ‐0.002 ± 0.013 |  |
| S-BIS measurements | | | | | | | |
| Whole skeletal muscle mass (kg) | No-Ex | 23.7 ± 6.9 | 23.8 ± 7.0 | 23.1 ± 6.5 | 0.1 ± 0.9 | ‐0.7 ± 0.7 | 0.257 |
|  | Low-Ex | 23.9 ± 7.3 | 23.7 ± 7.2 | 22.7 ± 6.6 | ‐0.1 ± 0.7 | ‐1.1 ± 1.0 |  |
|  | Moderate-Ex | 22.7 ± 7.0 | 22.9 ± 7.3 | 22.3 ± 6.9 | 0.2 ± 0.8 | ‐0.4 ± 0.8 |  |

Values are expressed as mean ± standard deviation. For the no-Ex (n = 17), low-Ex (n = 16), and moderate-Ex (n = 17) groups on MRI and DXA measurements, and the no-Ex (n = 15), low-Ex (n = 15) and moderate-Ex (n = 17) groups on S-BIS measurements, where 3 participants were not included for analysis because of unmeasurable data, there were no significant differences among the groups at baseline (one-way ANOVA). Ex, exercise; DXA, dual energy X-ray absorptiometry; BMD, bone mineral density; S-BIS, segmental bioelectrical impedance spectroscopy.

**Supplementary Table 3. Change rates of muscle quantity and quality by age in each group during the 24-week intervention.**

| Variable | Group | 50-59 years | 60-69 years | 70-79 years | Two-way ANOVA (*p*-value) | |
| --- | --- | --- | --- | --- | --- | --- |
|  |  |  |  |  | age | group*age |
| Thigh muscle CSA (%) | No-Ex | 1.1 ± 5.1 | ‐2.0 ± 6.0 | ‐1.3 ± 3.9 | 0.741 | 0.754 |
|  | Low-Ex | 3.2 ± 2.9 | 3.0 ± 7.5 | 6.0 ± 9.4 |  |  |
|  | Moderate-Ex | 4.5 ± 3.0 | 7.8 ± 4.3 | 9.1 ± 15.7 |  |  |
| Leg lean mass (%) | No-Ex | 0.7 ± 2.4 | 2.1 ± 3.6 | ‐2.1 ± 1.9 | 0.354 | 0.083 |
|  | Low-Ex | ‐0.4 ± 2.6 | 1.0 ± 2.5 | ‐1.5 ± 3.5 |  |  |
|  | Moderate-Ex | 2.8 ± 2.6 | 0.9 ± 2.9 | 3.5 ± 3.0 |  |  |
| IC resistance index (%) | No-Ex | ‐3.3 ± 8.2 | ‐7.5 ± 4.8 | ‐2.7 ± 7.2 | 0.682 | 0.559 |
|  | Low-Ex | ‐1.2 ± 4.0 | 0.2 ± 9.0 | ‐3.8 ± 3.5 |  |  |
|  | Moderate-Ex | 2.5 ± 4.2 | 2.1 ± 5.5 | ‐1.6 ± 8.6 |  |  |
| Resistance ratio (%) | No-Ex | ‐0.7 ± 9.9 | ‐0.1 ± 8.5 | 2.4 ± 6.7 | 0.920 | 0.549 |
|  | Low-Ex | 2.6 ± 4.0 | 6.3 ± 6.8 | 0.0 ± 7.8 |  |  |
|  | Moderate-Ex | 7.4 ± 5.6 | 3.8 ± 6.4 | 4.4 ± 9.3 |  |  |
| Membrane capacitance (%) | No-Ex | ‐4.9 ± 7.4 | ‐2.0 ± 12.1 | ‐4.1 ± 7.3 | 0.790 | 0.776 |
|  | Low-Ex | ‐1.3 ± 6.4 | 4.5 ± 11.9 | 0.9 ± 7.8 |  |  |
|  | Moderate-Ex | 9.4 ± 10.5 | 5.3 ± 13.6 | 3.5 ± 10.3 |  |  |
| Phase angle (%) | No-Ex | 0.1 ± 5.2 | ‐0.4 ± 6.6 | ‐0.5 ± 3.5 | 0.621 | 0.838 |
|  | Low-Ex | 2.6 ± 3.6 | 3.5 ± 6.0 | ‐0.1 ± 6.5 |  |  |
|  | Moderate-Ex | 6.2 ± 5.0 | 3.1 ± 5.0 | 4.1 ± 6.2 |  |  |

Values are expressed as mean ± standard deviation for the no-Ex (n = 6), low-Ex (n = 6) and moderate-Ex (n = 6) groups in 50-59 years, the no-Ex (n = 5), low-Ex (n = 5) and moderate-Ex (n = 6) groups in 60-69 years, and the no-Ex (n = 6), low-Ex (n = 5) and moderate-Ex (n = 5) groups in 70-79 years on muscle quantity and quality.

**Supplementary Table 4. Baseline characteristics in male participants**

|  | No-Ex  (n = 9) | Low-Ex  (n = 8) | Moderate-Ex  (n = 8) | *p*-value |
| --- | --- | --- | --- | --- |
| Age (years) | 62.9 ± 9.5 | 63.8 ± 7.6 | 63.4 ± 9.2 | 0.980 |
| Height (cm) | 168.9 ± 5.3 | 165.3 ± 3.0 | 171.1 ± 6.9 | 0.106 |
| Weight (kg) | 67.9 ± 10.1 | 67.7 ± 11.0 | 68.2 ± 9.1 | 0.995 |
| Thigh muscle CSA (cm^2^) | 116.1 ± 16.5 | 121.4 ± 23.9 | 118.8 ± 27.5 | 0.894 |
| Whole-body lean mass (kg) | 47.9 ± 6.6 | 49.6 ± 4.3 | 49.5 ± 5.8 | 0.780 |
| Hand grip strength (kg) | 37.2 ± 6.8 | 39.2 ± 6.3 | 37.7 ± 3.6 | 0.767 |

Values are expressed as mean ± standard deviation. There was no significant difference among the groups in baseline data (ANOVA). Ex, exercise; CSA, cross-sectional area.

**Supplementary Table 5. Baseline characteristics in female participants**

|  | No-Ex  (n = 8) | Low-Ex  (n = 8) | Moderate-Ex  (n = 9) | *p*-value |
| --- | --- | --- | --- | --- |
| Age (years) | 64.3 ± 7.8 | 63.5 ± 9.1 | 63.7 ± 7.9 | 0.982 |
| Height (cm) | 154.6 ± 1.9 | 154.1 ± 5.2 | 153.0 ± 4.2 | 0.690 |
| Weight (kg) | 49.5 ± 6.6 | 51.7 ± 6.1 | 51.1 ± 6.4 | 0.788 |
| Thigh muscle CSA (cm^2^) | 83.7 ± 12.4 | 83.7 ± 9.8 | 86.4 ± 11.5 | 0.848 |
| Whole-body lean mass (kg) | 33.4 ± 3.5 | 33.0 ± 1.3 | 33.3 ± 2.8 | 0.949 |
| Hand grip strength (kg) | 24.9 ± 3.5 | 21.7 ± 4.1 | 21.9 ± 4.4 | 0.231 |

Values are expressed as mean ± standard deviation. There was no significant difference among the groups in baseline data (ANOVA). Ex, exercise; CSA, cross-sectional area.

**Supplementary Table 6. Muscle quantity, quality and strength in men by groups during the intervention**

| Variable | Group | Baseline | 12 weeks | 24 weeks | Change  (Δ 12 weeks) | Change  (Δ 24 weeks) | Two-way ANOVA  (group*time)  *p*-value |
| --- | --- | --- | --- | --- | --- | --- | --- |
| MRI measurements | | | | | | | |
| Thigh  CSA (cm^2^) | No-Ex | 116.1 ± 16.5 | 111.6 ± 15.1 | 113.9 ± 13.2 | ‐4.5 ± 6.2 | ‐2.3 ± 6.7 | 0.004  ** |
|  | Low-Ex | 121.4 ± 23.9 | 123.4 ± 22.4 | 127.0 ± 25.0 | 2.0 ± 8.6 | 5.7 ± 9.3 |  |
|  | Moderate-Ex | 118.8 ± 27.5 | 120.8 ± 27.9 | 129.8 ± 24.8^§§^ | 2.1 ± 3.5 | 11.0 ± 7.8 |  |
| Quadriceps  CSA (cm^2^) | No-Ex | 59.4 ± 9.0 | 57.0 ± 8.0 | 57.2 ± 7.7 | ‐2.4 ± 4.1 | ‐2.1 ± 5.0  * | 0.005  * |
|  | Low-Ex | 61.2 ± 11.5 | 64.3 ± 10.7 | 65.0 ± 12.1 | 3.1 ± 6.1 | 3.7 ± 5.9 |  |
|  | Moderate-Ex | 58.4 ± 15.1 | 61.7 ± 14.2^§^ | 64.4 ± 13.0^§§^ | 3.3 ± 3.1 | 6.0 ± 5.0 |  |
| Vastus lateralis  CSA (cm^2^) | No-Ex | 18.9 ± 3.3 | 17.5 ± 3.2^§^ | 19.2 ± 3.1 | ‐1.3 ± 0.3  ** | 0.3 ± 1.7 | 0.002  ** |
|  | Low-Ex | 19.6 ± 4.2 | 19.8 ± 4.1 | 21.2 ± 4.8 | 0.2 ± 2.2 | 1.6 ± 2.5 |  |
|  | Moderate-Ex | 19.5 ± 5.8 | 20.9 ± 5.1^§^ | 23.3 ± 5.3^§§^ | 1.4 ± 1.4 | 3.8 ± 1.4 |  |
| Subcutaneous fat  CSA (cm^2^) | No-Ex | 43.7 ± 11.1 | 44.8 ± 11.4 | 41.9 ± 11.3 | 1.1 ± 5.5 | ‐1.8 ± 2.9 | 0.359 |
|  | Low-Ex | 44.5 ± 12.3 | 41.6 ± 11.5 | 38.7 ± 12.8 | ‐3.0 ± 4.1 | ‐5.8 ± 3.4 |  |
|  | Moderate-Ex | 49.5 ± 12.3 | 49.9 ± 9.0 | 41.0 ± 10.0 | 0.4 ± 10.5 | ‐8.5 ± 16.2 |  |
| IMAT (cm^2^) | No-Ex | 4.4 ± 2.1 | 4.7 ± 2.5 | 3.9 ± 2.2 | 0.3 ± 0.8 | ‐0.5 ± 0.8 | 0.327 |
|  | Low-Ex | 5.3 ± 2.2 | 4.7 ± 1.8 | 4.2 ± 1.3 | ‐0.6 ± 0.8 | ‐1.1 ± 1.2 |  |
|  | Moderate-Ex | 5.6 ± 1.9 | 5.9 ± 2.4 | 4.6 ± 1.4 | 0.3 ± 1.6 | ‐1.0 ± 1.2 |  |
| IMAT / Thigh muscle CSA | No-Ex | 0.038 ± 0.016 | 0.043 ± 0.021 | 0.034 ± 0.019 | 0.005 ± 0.010 | ‐0.004 ± 0.009 | 0.170 |
|  | Low-Ex | 0.044 ± 0.016 | 0.038 ± 0.012 | 0.033 ± 0.008 | ‐0.005 ± 0.008 | ‐0.010 ± 0.012 |  |
|  | Moderate-Ex | 0.049 ± 0.018 | 0.049 ± 0.015 | 0.036 ± 0.010 | 0.000 ± 0.013 | ‐0.014 ± 0.015 |  |
| DXA measurements | | | | | | | |
| Leg lean mass  (kg) | No-Ex | 15.51 ± 2.38 | 15.84 ± 2.60 | 15.76 ± 2.54 | 0.34 ± 0.52 | 0.26 ± 0.54 | 0.925 |
|  | Low-Ex | 16.63 ± 1.90 | 16.76 ± 1.82 | 16.80 ± 1.75 | 0.13 ± 0.36 | 0.17 ± 0.41 |  |
|  | Moderate-Ex | 16.56 ± 2.44 | 16.70 ± 2.39 | 16.76 ± 2.69 | 0.14 ± 0.65 | 0.20 ± 0.51 |  |
| Arm lean mass  (kg) | No-Ex | 5.64 ± 0.79 | 5.67 ± 0.83 | 5.63 ± 0.83 | 0.03 ± 0.17 | ‐0.01 ± 0.12 | 0.965 |
|  | Low-Ex | 5.91 ± 0.70 | 5.98 ± 0.69 | 5.93 ± 0.74 | 0.07 ± 0.13 | 0.02 ± 0.10 |  |
|  | Moderate-Ex | 5.73 ± 0.88 | 5.77 ± 0.91 | 5.76 ± 0.95 | 0.04 ± 0.17 | 0.03 ± 0.15 |  |
| Whole-body lean  mass (kg) | No-Ex | 47.9 ± 6.6 | 48.3 ± 6.6 | 47.8 ± 6.3 | 0.5 ± 0.8 | 0.0 ± 0.8 | 0.123 |
|  | Low-Ex | 49.6 ± 4.3 | 49.9 ± 3.9 | 49.8 ± 4.5 | 0.3 ± 0.8 | 0.3 ± 1.0 |  |
|  | Moderate-Ex | 49.5 ± 5.8 | 50.4 ± 6.0 | 50.5 ± 6.1 | 0.9 ± 0.9 | 1.0 ± 0.8 |  |
| S-BIS measurements of the thigh | | | | | | | |
| IC resistance index  (cm^2^/Ω) | No-Ex | 13.1 ± 2.2 | 12.3 ± 1.8 | 12.3 ± 1.9 | ‐0.9 ± 0.9 | ‐0.8 ± 0.7 | 0.077 |
|  | Low-Ex | 14.7 ± 3.1 | 14.2 ± 3.5 | 14.3 ± 2.9 | ‐0.4 ± 0.9 | ‐0.4 ± 0.9 |  |
|  | Moderate-Ex | 14.4 ± 3.2 | 14.6 ± 3.5 | 14.5 ± 3.5 | 0.2 ± 0.9 | 0.1 ± 0.9 |  |
| EC resistance index  (cm^2^/Ω) | No-Ex | 28.5 ± 4.6 | 28.6 ± 4.5 | 26.7 ± 3.6 | 0.1 ± 1.6 | ‐1.7 ± 1.5 | 0.877 |
|  | Low-Ex | 30.3 ± 2.9 | 30.0 ± 2.7 | 28.4 ± 2.2 | ‐0.3 ± 1.6 | ‐2.0 ± 1.1 |  |
|  | Moderate-Ex | 30.2 ± 2.9 | 30.3 ± 4.3 | 29.0 ± 3.7 | 0.1 ± 1.6 | ‐1.3 ± 1.1 |  |
| Resistance ratio | No-Ex | 0.46 ± 0.05 | 0.43 ± 0.03 | 0.46 ± 0.05 | ‐0.03 ± 0.04 | 0.00 ± 0.04 | 0.101 |
|  | Low-Ex | 0.48 ± 0.08 | 0.47 ± 0.08 | 0.50 ± 0.08 | ‐0.01 ± 0.02 | 0.02 ± 0.02 |  |
|  | Moderate-Ex | 0.47 ± 0.07 | 0.48 ± 0.06 | 0.50 ± 0.08 | 0.00 ± 0.02 | 0.02 ± 0.03 |  |
| Characteristic frequency  (kHz) | No-Ex | 44.6 ± 5.0 | 45.3 ± 5.2 | 43.3 ± 4.7 | 0.7 ± 5.3 | ‐1.3 ± 3.3 | 0.356 |
|  | Low-Ex | 44.8 ± 6.7 | 45.8 ± 6.3 | 43.3 ± 7.7 | 1.0 ± 4.6 | ‐1.5 ± 3.5 |  |
|  | Moderate-Ex | 48.2 ± 9.5 | 45.9 ± 7.9 | 43.9 ± 8.8 | ‐2.3 ± 2.7 | ‐4.3 ± 2.7 |  |
| Membrane capacitance  (nF) | No-Ex | 13.7 ± 2.1 | 12.9 ± 1.8 | 13.3 ± 1.9 | ‐0.8 ± 1.5 | ‐0.4 ± 1.1 | 0.038  * |
|  | Low-Ex | 15.7 ± 3.6 | 15.0 ± 4.0 | 15.7 ± 3.8 | ‐0.7 ± 1.5 | 0.0 ± 1.1 |  |
|  | Moderate-Ex | 14.1 ± 4.4 | 14.8 ± 4.6 | 15.3 ± 5.1^§^ | 0.7 ± 1.0 | 1.3 ± 1.4 |  |
| Phase angle  (deg) | No-Ex | 6.6 ± 0.6 | 6.3 ± 0.4^§^ | 6.6 ± 0.6 | ‐0.3 ± 0.3 | 0.0 ± 0.3  * | 0.036 |
|  | Low-Ex | 7.0 ± 0.9 | 6.8 ± 0.9^§^ | 7.2 ± 1.0 | ‐0.2 ± 0.2 | 0.2 ± 0.3 |  |
|  | Moderate-Ex | 6.7 ± 0.9 | 6.8 ± 0.8 | 7.0 ± 1.0^§^ | 0.1 ± 0.3 | 0.3 ± 0.3 |  |
| Muscle strength measurements | | | | | | | |
| Hand grip strength  (kg) | No-Ex | 37.2 ± 6.8 | 38.4 ± 7.3 | 38.8 ± 7.5 | 1.2 ± 2.2 | 1.6 ± 1.6 | 0.760 |
|  | Low-Ex | 39.2 ± 6.3 | 39.6 ± 6.1 | 39.9 ± 6.7 | 0.4 ± 1.6 | 0.7 ± 1.5 |  |
|  | Moderate-Ex | 37.7 ± 3.6 | 37.8 ± 4.7 | 38.5 ± 4.4 | 0.2 ± 2.5 | 0.8 ± 1.8 |  |

Values are expressed as mean ± standard deviation. For the no-Ex (n = 9), low-Ex (n = 8), and moderate-Ex (n = 8) groups on MRI, DXA, S-BIS and muscle strength measurements, there were no significant differences among the groups at baseline (one-way ANOVA). **p* < 0.05 and ***p* < 0.01 compared among the groups (Tukey's test), ^§^*p* < 0.05 and ^§§^*p* < 0.01 compared with values at baseline (Dunnett's test). Ex, exercise; MRI, magnetic resonance imaging; CSA, cross-sectional area; IMAT, intermuscular adipose tissue; DXA, dual energy X-ray absorptiometry; S-BIS, segmental bioelectrical impedance spectroscopy; IC, intracellular; EC, extracellular; resistance ratio, resistance ratio of intracellular to extracellular fluid.

**Supplementary Table 7. Muscle quantity, quality and strength in women by groups during the intervention**

| Variable | Group | Baseline | 12 weeks | 24 weeks | Change  (Δ 12 weeks) | Change  (Δ 24 weeks) | Two-way ANOVA  (group*time)  *p*-value |
| --- | --- | --- | --- | --- | --- | --- | --- |
| MRI measurements | | | | | | | |
| Thigh  CSA (cm^2^) | No-Ex | 83.7 ± 12.4 | 83.5 ± 12.7 | 83.9 ± 13.3 | ‐0.2 ± 3.7 | 0.2 ± 3.6 | 0.150 |
|  | Low-Ex | 83.7 ± 9.8 | 82.3 ± 10.3 | 86.3 ± 9.6 | ‐1.3 ± 3.3 | 2.6 ± 2.9 |  |
|  | Moderate-Ex | 86.4 ± 11.5 | 88.2 ± 11.5 | 89.5 ± 10.4 | 1.8 ± 2.4 | 3.1 ± 4.8 |  |
| Quadriceps  CSA (cm^2^) | No-Ex | 42.6 ± 6.1 | 42.7 ± 6.1 | 41.5 ± 7.2 | 0.1 ± 1.8 | ‐1.1 ± 1.9 | 0.082 |
|  | Low-Ex | 42.1 ± 5.9 | 42.4 ± 6.4 | 43.0 ± 5.8 | 0.2 ± 1.4 | 0.9 ± 1.0 |  |
|  | Moderate-Ex | 43.9 ± 5.1 | 46.0 ± 6.1 | 45.2 ± 4.8 | 2.1 ± 1.9 | 1.3 ± 3.4 |  |
| Vastus lateralis  CSA (cm^2^) | No-Ex | 14.9 ± 1.9 | 14.9 ± 2.3 | 14.8 ± 2.4 | 0.1 ± 1.7 | 0.0 ± 1.1 | 0.025 |
|  | Low-Ex | 14.5 ± 2.5 | 13.9 ± 2.6 | 15.4 ± 2.5 | ‐0.6 ± 1.5 | 0.9 ± 1.3 |  |
|  | Moderate-Ex | 16.2 ± 2.6 | 17.4 ± 3.5^§^ | 17.3 ± 2.4 | 1.3 ± 1.6 | 1.1 ± 1.4 |  |
| Subcutaneous fat  CSA (cm^2^) | No-Ex | 67.6 ± 15.3 | 59.9 ± 18.7 | 62.3 ± 14.4 | ‐7.6 ± 7.6 | ‐5.3 ± 5.5 | 0.518 |
|  | Low-Ex | 61.1 ± 61.4 | 61.4 ± 16.0 | 60.7 ± 15.9 | 0.2 ± 11.8 | ‐0.4 ± 11.6 |  |
|  | Moderate-Ex | 63.0 ± 14.8 | 55.8 ± 14.4 | 59.1 ± 9.5 | ‐7.2 ± 8.4 | ‐3.9 ± 13.2 |  |
| IMAT (cm^2^) | No-Ex | 4.3 ± 1.8 | 4.2 ± 1.2 | 3.9 ± 1.4 | 0.3 ± 1.1 | ‐0.4 ± 1.0 | 0.570 |
|  | Low-Ex | 4.5 ± 1.5 | 4.3 ± 1.6 | 3.4 ± 1.7 | ‐0.3 ± 0.5 | ‐1.1 ± 0.5 |  |
|  | Moderate-Ex | 5.3 ± 1.2 | 4.9 ± 1.5 | 4.3 ± 1.6 | ‐0.4 ± 0.9 | ‐0.9 ± 0.9 |  |
| IMAT / Thigh muscle CSA | No-Ex | 0.054 ± 0.030 | 0.052 ± 0.017 | 0.048 ± 0.022 | 0.004 ± 0.012 | ‐0.006 ± 0.013 | 0.484 |
|  | Low-Ex | 0.055 ± 0.018 | 0.052 ± 0.021 | 0.040 ± 0020 | ‐0.002 ± 0.006 | ‐0.014 ± 0.005 |  |
|  | Moderate-Ex | 0.062 ± 0.015 | 0.056 ± 0.018 | 0.049 ± 0.020 | ‐0.005 ± 0.010 | ‐0.013 ± 0.011 |  |
| DXA measurements | | | | | | | |
| Leg lean mass  (kg) | No-Ex | 10.97 ± 1.44 | 10.71 ± 1.24^§^ | 10.80 ± 1.36 | ‐0.26 ± 0.31  * | ‐0.17 ± 0.17  * | <0.001  ** |
|  | Low-Ex | 11.11 ± 0.78 | 11.24 ± 0.94 | 10.91 ± 0.76 | 0.13 ± 0.34 | ‐0.20 ± 0.26 |  |
|  | Moderate-Ex | 10.61 ± 0.92 | 10.75 ± 0.96 | 10.96 ± 0.86^§§^ | 0.14 ± 0.15 | 0.35 ± 0.26  ** |  |
| Arm lean mass  (kg) | No-Ex | 3.35 ± 0.43 | 3.32 ± 0.38 | 3.33 ± 0.37 | ‐0.04 ± 0.06 | ‐0.03 ± 0.10 | 0.650 |
|  | Low-Ex | 3.40 ± 0.45 | 3.43 ± 0.53 | 3.45 ± 0.49 | 0.03 ± 0.16 | 0.04 ± 0.11 |  |
|  | Moderate-Ex | 3.31 ± 0.46 | 3.33 ± 0.46 | 3.37 ± 0.38 | 0.02 ± 0.18 | 0.06 ± 0.13 |  |
| Whole-body lean  mass (kg) | No-Ex | 33.4 ± 3.5 | 33.3 ± 3.4 | 33.4 ± 3.5 | ‐0.1 ± 0.8 | 0.0 ± 0.5 | 0.032 |
|  | Low-Ex | 33.0 ± 1.3 | 33.8 ± 2.0^§^ | 33.3 ± 1.7 | 0.8 ± 0.9 | 0.3 ± 0.5 |  |
|  | Moderate-Ex | 33.3 ± 2.8 | 33.8 ± 2.7 | 34.0 ± 2.9^§§^ | 0.4 ± 0.4 | 0.7 ± 0.7 |  |
| S-BIS measurements of the thigh | | | | | | | |
| IC resistance index  (cm^2^/Ω) | No-Ex | 8.3 ± 1.8 | 8.5 ± 2.0 | 8.2 ± 2.1 | 0.2 ± 0.8 | ‐0.1 ± 0.8 | 0.025 |
|  | Low-Ex | 8.8 ± 1.2 | 8.2 ± 1.1^§^ | 8.7 ± 1.2 | ‐0.5 ± 0.6 | 0.0 ± 0.5 |  |
|  | Moderate-Ex | 8.4 ± 1.0 | 8.6 ± 1.0 | 8.5 ± 0.8 | 0.2 ± 0.4 | 0.1 ± 0.5 |  |
| EC resistance index  (cm^2^/Ω) | No-Ex | 21.8 ± 3.1 | 21.7 ± 2.9 | 21.1 ± 3.0 | ‐0.1 ± 1.2 | ‐0.7 ± 0.5 | 0.995 |
|  | Low-Ex | 20.9 ± 1.3 | 20.9 ± 0.8 | 20.4 ± 1.1 | 0.0 ± 1.0 | ‐0.5 ± 1.0 |  |
|  | Moderate-Ex | 20.4 ± 1.8 | 20.2 ± 1.6 | 19.7 ± 1.6 | ‐0.1 ± 1.1 | ‐0.7 ± 0.9 |  |
| Resistance ratio | No-Ex | 0.38 ± 0.07 | 0.39 ± 0.07 | 0.38 ± 0.07 | 0.01 ± 0.03 | 0.00 ± 0.03 | 0.044 |
|  | Low-Ex | 0.42 ± 0.05 | 0.39 ± 0.05 | 0.43 ± 0.05 | ‐0.03 ± 0.03 | 0.01 ± 0.03 |  |
|  | Moderate-Ex | 0.41 ± 0.05 | 0.43 ± 0.06 | 0.44 ± 0.04 | 0.01 ± 0.02 | 0.02 ± 0.03 |  |
| Characteristic frequency  (kHz) | No-Ex | 58.1 ± 6.4 | 59.8 ± 7.2 | 59.8 ± 6.3 | 1.7 ± 3.2 | 1.8 ± 2.6 | 0.583 |
|  | Low-Ex | 52.0 ± 6.7 | 52.6 ± 7.0 | 50.4 ± 6.1 | 0.6 ± 4.2 | ‐1.6 ± 4.2 |  |
|  | Moderate-Ex | 53.3 ± 9.1 | 53.5 ± 8.4 | 51.5 ± 7.4 | 0.1 ± 5.5 | ‐1.9 ± 6.0 |  |
| Membrane capacitance  (nF) | No-Ex | 8.1 ± 2.1 | 8.0 ± 1.9 | 7.7 ± 2.0 | ‐0.1 ± 0.6 | ‐0.4 ± 0.8 | 0.195 |
|  | Low-Ex | 9.4 ± 2.1 | 8.9 ± 2.1 | 9.5 ± 1.8 | ‐0.5 ± 0.9 | 0.1 ± 0.8 |  |
|  | Moderate-Ex | 9.4 ± 1.9 | 9.5 ± 1.9 | 9.7 ± 1.8 | 0.1 ± 0.7 | 0.3 ± 1.2 |  |
| Phase angle  (deg) | No-Ex | 5.5 ± 0.7 | 5.5 ± 0.7 | 5.5 ± 0.8 | 0.0 ± 0.2 | 0.0 ± 0.3 | 0.125 |
|  | Low-Ex | 5.9 ± 0.7 | 5.7 ± 0.7 | 6.0 ± 0.6 | ‐0.2 ± 0.3 | 0.1 ± 0.4 |  |
|  | Moderate-Ex | 5.9 ± 0.6 | 5.9 ± 0.8 | 6.1 ± 0.7 | 0.1 ± 0.3 | 0.3 ± 0.3 |  |
| Muscle strength measurements | | | | | | | |
| Hand grip strength  (kg) | No-Ex | 24.9 ± 3.5 | 24.0 ± 3.2 | 24.7 ± 3.7 | ‐0.8 ± 1.4 | ‐0.2 ± 1.8 | 0.272 |
|  | Low-Ex | 21.7 ± 4.1 | 22.5 ± 3.7 | 24.1 ± 4.1 | 0.8 ± 2.3 | 2.4 ± 3.3 |  |
|  | Moderate-Ex | 21.9 ± 4.4 | 22.8 ± 3.8 | 23.4 ± 3.8 | 0.9 ± 3.3 | 1.4 ± 3.9 |  |

Values are expressed as mean ± standard deviation. For the no-Ex (n = 8), low-Ex (n = 8), and moderate-Ex (n = 9) groups on MRI, DXA and muscle strength measurements, and the no-Ex (n = 7), low-Ex (n = 7) and moderate-Ex (n = 9) groups on S-BIS measurements, where 2 participants were not included for analysis because of unmeasurable data, there were no significant differences among the groups at baseline (one-way ANOVA). **p* < 0.05 and ***p* < 0.01 compared among the groups (Tukey's test), ^§^*p* < 0.05 and ^§§^*p* < 0.01 compared with values at baseline (Dunnett's test). Ex, exercise; MRI, magnetic resonance imaging; CSA, cross-sectional area; IMAT, intermuscular adipose tissue; DXA, dual energy X-ray absorptiometry; S-BIS, segmental bioelectrical impedance spectroscopy; IC, intracellular; EC, extracellular; resistance ratio, resistance ratio of intracellular to extracellular fluid.

**Supplementary Table 8. 1-RM in men by groups during the intervention**

| Variables | Group |  | | | | | | |
| --- | --- | --- | --- | --- | --- | --- | --- | --- |
| Leg extension (kg) |  | Values at each time point | | | | | | |
|  |  | Baseline | 4 weeks | 8 weeks | 12 weeks | 16 weeks | 20 weeks | 24 weeks |
|  | low-Ex | 62.8 ± 9.5 | 72.6 ± 11.6^§^ | 78.9 ± 14.8^§§^ | 79.3 ± 17.0^§§^ | 81.2 ± 15.4^§§^ | 83.8 ± 14.6^§§^ | 85.7 ± 14.6^§§^ |
|  | moderate-Ex | 53.0 ± 20.9 | 70.4 ± 15.2^§§^ | 72.2 ± 15.8^§§^ | 77.9 ± 13.4^§§^ | 81.6 ± 14.1^§§^ | 87.5 ± 14.8^§§^ | 90.2 ± 19.3^§§^ |
|  |  | Change | | | | | | |
|  |  |  | Δ 4 weeks | Δ 8 weeks | Δ 12 weeks | Δ 16 weeks | Δ 20 weeks | Δ 24 weeks |
|  | low-Ex |  | 9.8 ± 11.8 | 16.1 ± 13.3 | 16.5 ± 14.8 | 18.4 ± 12.7 | 21.0 ± 12.7 | 22.9 ± 12.7 |
|  | moderate-Ex |  | 13.1 ± 7.4 | 19.2 ± 11.1 | 24.9 ± 11.2 | 28.6 ± 11.2 | 34.4 ± 12.8 | 37.2 ± 13.9  * |
| Leg curl (kg) |  | Values at each time point | | | | | | |
|  |  | Baseline | 4 weeks | 8 weeks | 12 weeks | 16 weeks | 20 weeks | 24 weeks |
|  | low-Ex | 28.7 ± 6.1 | 32.4 ± 6.4^§^ | 34.5 ± 5.4^§§^ | 34.4 ± 5.5^§§^ | 35.8 ± 5.3^§§^ | 36.9 ± 4.5^§§^ | 39.9 ± 4.5^§§^ |
|  | moderate-Ex | 30.8 ± 8.9 | 36.2 ± 8.6^§§^ | 36.2 ± 9.2^§§^ | 39.3 ± 8.7^§§^ | 41.1 ± 9.3^§§^ | 41.4 ± 11.6^§§^ | 43.1 ± 13.2^§§^ |
|  |  | Change | | | | | | |
|  |  |  | Δ 4 weeks | Δ 8 weeks | Δ 12 weeks | Δ 16 weeks | Δ 20 weeks | Δ 24 weeks |
|  | low-Ex |  | 3.7 ± 4.5 | 5.8 ± 4.9 | 7.1 ± 6.4 | 7.1 ± 6.1 | 8.2 ± 5.3 | 11.2 ± 5.7 |
|  | moderate-Ex |  | 4.4 ± 3.1 | 5.4 ± 1.8 | 8.5 ± 2.2 | 10.3 ± 1.8 | 10.6 ± 1.8 | 12.3 ± 6.1 |
| Leg press (kg) |  | Values at each time point | | | | | | |
|  |  | Baseline | 4 weeks | 8 weeks | 12 weeks | 16 weeks | 20 weeks | 24 weeks |
|  | low-Ex | 164.2 ± 42.3 | 202.6 ± 34.1^§§^ | 209.2 ± 33.9^§§^ | 207.1 ± 36.5^§§^ | 206.7 ± 54.9^§§^ | 198.8 ± 38.2^§§^ | 210.0 ± 42.4^§§^ |
|  | moderate-Ex | 163.4 ± 45.2 | 199.4 ± 52.2^§§^ | 202.5 ± 53.4^§§^ | 215.0 ± 60.6^§§^ | 225.7 ± 52.8^§§^ | 230.0 ± 52.0^§§^ | 243.8 ± 69.2^§§^ |
|  |  | Change | | | | | | |
|  |  |  | Δ 4 weeks | Δ 8 weeks | Δ 12 weeks | Δ 16 weeks | Δ 20 weeks | Δ 24 weeks |
|  | low-Ex |  | 38.4 ± 22.9 | 45.0 ± 22.5 | 42.9 ± 23.0 | 42.5 ± 45.6 | 34.6 ± 37.9 | 45.8 ± 36.4 |
|  | moderate-Ex |  | 29.3 ± 16.2 | 39.1 ± 18.6 | 51.6 ± 23.8 | 62.2 ± 18.3 | 66.6 ± 21.5 | 80.4 ± 31.5 |
| Chest press (kg) |  | Values at each time point | | | | | | |
|  |  | Baseline | 4 weeks | 8 weeks | 12 weeks | 16 weeks | 20 weeks | 24 weeks |
|  | low-Ex | 52.5 ± 18.0 | 59.6 ± 17.6^§^ | 62.9 ± 18.0^§§^ | 64.7 ± 18.7^§§^ | 65.8 ± 15.9^§§^ | 68.1 ± 18.4^§§^ | 68.9 ± 18.7^§§^ |
|  | moderate-Ex | 50.2 ± 10.4 | 61.4 ± 9.6^§§^ | 61.9 ± 12.1^§§^ | 66.3 ± 12.9^§§^ | 68.0 ± 11.4^§§^ | 69.9 ± 10.5^§§^ | 72.1 ± 13.6^§§^ |
|  |  | Change | | | | | | |
|  |  |  | Δ 4 weeks | Δ 8 weeks | Δ 12 weeks | Δ 16 weeks | Δ 20 weeks | Δ 24 weeks |
|  | low-Ex |  | 7.1 ± 6.3 | 10.4 ± 10.0 | 12.2 ± 8.1 | 13.4 ± 9.6 | 15.6 ± 8.7 | 16.5 ± 8.7 |
|  | moderate-Ex |  | 9.9 ± 6.8 | 11.7 ± 5.9 | 16.1 ± 6.1 | 17.8 ± 6.3 | 19.7 ± 5.8 | 22.0 ± 7.9 |

Values are expressed as mean ± standard deviation. For the low-Ex (n = 8), and moderate-Ex (n = 8) groups on 1RM tests, there were no significant differences among the groups at baseline (student’s *t*-test). **p* < 0.05 compared among the groups (Student's *t*-test), ^§^*p* < 0.05 and ^§§^*p* < 0.01 compared with values at baseline (Dunnett's test). 1-RM, 1-repetition maximum.

**Supplementary Table 9. 1-RM in women by groups during the intervention**

| Variables | Group |  | | | | | | |
| --- | --- | --- | --- | --- | --- | --- | --- | --- |
| Leg extension (kg) |  | Values at each time point | | | | | | |
|  |  | Baseline | 4 weeks | 8 weeks | 12 weeks | 16 weeks | 20 weeks | 24 weeks |
|  | low-Ex | 35.6 ± 10.0 | 49.0 ± 9.9^§§^ | 50.3 ± 10.3^§§^ | 52.9 ± 10.6^§§^ | 54.2 ± 10.4^§§^ | 54.1 ± 9.9^§§^ | 55.5 ± 15.3^§§^ |
|  | moderate-Ex | 41.2 ± 11.4 | 50.1 ± 10.4^§§^ | 51.8 ± 9.0^§§^ | 53.8 ± 9.9^§§^ | 55.8 ± 9.7^§§^ | 57.2 ± 8.1^§§^ | 59.4 ± 8.9^§§^ |
|  |  | Change | | | | | | |
|  |  |  | Δ 4 weeks | Δ 8 weeks | Δ 12 weeks | Δ 16 weeks | Δ 20 weeks | Δ 24 weeks |
|  | low-Ex |  | 13.4 ± 5.4 | 14.7 ± 5.7 | 17.3 ± 6.0 | 18.6 ± 6.5 | 18.5 ± 6.2 | 19.9 ± 10.6 |
|  | moderate-Ex |  | 8.9 ± 4.9 | 10.6 ± 5.8 | 13.9 ± 5.9 | 14.6 ± 7.3 | 16.0 ± 8.6 | 18.2 ± 10.0 |
| Leg curl (kg) |  | Values at each time point | | | | | | |
|  |  | Baseline | 4 weeks | 8 weeks | 12 weeks | 16 weeks | 20 weeks | 24 weeks |
|  | low-Ex | 15.2 ± 4.2 | 21.2 ± 3.0^§§^ | 22.0 ± 3.2^§§^ | 23.6 ± 2.7^§§^ | 23.6 ± 3.0^§§^ | 25.1 ± 4.0^§§^ | 26.1 ± 5.5^§§^ |
|  | moderate-Ex | 18.9 ± 4.3 | 21.6 ± 5.1^§§^ | 22.8 ± 5.1^§§^ | 23.0 ± 4.3^§§^ | 23.7 ± 4.5^§§^ | 24.7 ± 4.7^§§^ | 25.6 ± 4.4^§§^ |
|  |  | Change | | | | | | |
|  |  |  | Δ 4 weeks | Δ 8 weeks | Δ 12 weeks | Δ 16 weeks  * | Δ 20 weeks  * | Δ 24 weeks  * |
|  | low-Ex |  | 5.9 ± 2.8 | 6.8 ± 2.5 | 8.4 ± 2.6  ** | 8.3 ± 2.3 | 9.9 ± 3.1 | 10.8 ± 3.6 |
|  | moderate-Ex |  | 2.7 ± 3.5 | 3.9 ± 3.5 | 4.1 ± 3.2 | 4.8 ± 3.3 | 5.8 ± 3.9 | 6.6 ± 4.3 |
| Leg press (kg) |  | Values at each time point | | | | | | |
|  |  | Baseline | 4 weeks | 8 weeks | 12 weeks | 16 weeks  * | 20 weeks | 24 weeks |
|  | low-Ex | 111.5 ± 17.2 | 122.6 ± 13.8 | 128.2 ± 23.0^§^ | 133.3 ± 27.1^§§^ | 137.0 ± 27.3^§§^ | 143.7 ± 28.8^§§^ | 148.6 ± 22.8^§§^ |
|  | moderate-Ex | 123.4 ± 18.7 | 147.7 ± 28.7^§§^ | 152.7 ± 28.5^§§^ | 166.6 ± 26.9^§§^ | 162.6 ± 27.7^§§^ | 171.4 ± 30.0^§§^ | 176.7 ± 39.9^§§^ |
|  |  | Change | | | | | | |
|  |  |  | Δ 4 weeks  * | Δ 8 weeks | Δ 12 weeks | Δ 16 weeks | Δ 20 weeks | Δ 24 weeks |
|  | low-Ex |  | 11.1 ± 8.4 | 16.7 ± 14.1  * | 21.8 ± 19.1 | 25.5 ± 19.9 | 32.2 ± 17.3  * | 37.1 ± 14.0 |
|  | moderate-Ex |  | 24.2 ± 14.6 | 29.3 ± 16.9 | 43.1 ± 17.9 | 39.2 ± 15.0 | 48.0 ± 18.8 | 53.3 ± 33.8 |
| Chest press (kg) |  | Values at each time point | | | | | | |
|  |  | Baseline | 4 weeks | 8 weeks | 12 weeks | 16 weeks | 20 weeks | 24 weeks |
|  | low-Ex | 28.7 ± 4.9 | 33.4 ± 5.3^§§^ | 33.8 ± 5.7^§§^ | 36.2 ± 5.4^§§^ | 36.3 ± 5.3^§§^ | 36.4 ± 6.3^§§^ | 38.0 ± 7.1^§§^ |
|  | moderate-Ex | 28.8 ± 4.7 | 32.6 ± 5.7 | 33.6 ± 5.7^§^ | 35.0 ± 7.7^§§^ | 36.4 ± 6.4^§§^ | 37.6 ± 6.9^§§^ | 39.7 ± 7.4^§§^ |
|  |  | Change | | | | | | |
|  |  |  | Δ 4 weeks | Δ 8 weeks | Δ 12 weeks | Δ 16 weeks | Δ 20 weeks | Δ 24 weeks |
|  | low-Ex |  | 4.7 ± 2.5 | 5.1 ± 3.0 | 7.5 ± 2.6 | 7.6 ± 2.2 | 7.7 ± 4.8 | 9.3 ± 4.4 |
|  | moderate-Ex |  | 3.7 ± 4.4 | 4.7 ± 4.4 | 6.1 ± 6.6 | 7.6 ± 6.0 | 8.8 ± 6.5 | 10.9 ± 6.3 |

Values are expressed as mean ± standard deviation. For the low-Ex (n = 8), and moderate-Ex (n = 9) groups on 1RM tests, there were no significant differences among the groups at baseline (student’s *t*-test). **p* < 0.05 compared among the groups (Student's *t*-test), ^§^*p* < 0.05 and ^§§^*p* < 0.01 compared with values at baseline (Dunnett's test). 1-RM, 1-repetition maximum.
